# Targeting neuroinflammation: 3-monothiopomalidomide a new drug candidate to mitigate traumatic brain injury and neurodegeneration

**DOI:** 10.1186/s12929-025-01150-w

**Published:** 2025-06-16

**Authors:** Shih Chang Hsueh, Pathik Parekh, Buyandelger Batsaikhan, Neil Vargesson, David Tweedie, Weiming Luo, Chirag N. Patel, Dong Liu, Ross A. McDevitt, Abdul Mannan Baig, Yu Kyung Kim, Sun Kim, Inho Hwang, Juwan Kim, Mee Youn Lee, Anna R. Carta, Warren R. Selman, Barry J. Hoffer, Dong Seok Kim, Nigel H. Greig

**Affiliations:** 1 Drug Design & Development Section, Translational Gerontology Branch, Intramural Research Program National Institute on Aging, NIH, Baltimore, MD 21224 USA; 2 School of Medicine, Medical Sciences and Nutrition, Institute of Medical Sciences, University of Aberdeen, Aberdeen, AB25 2ZD Scotland, UK; 3 Comparative Medicine Section, National Institute on Aging, Baltimore, MD USA; 4 St. George Hospital, 83043 Bad Aibling, Germany; 5 Aevis Bio Inc., Daejeon, 34141 Republic of Korea; 6 Department of Biomedical Sciences, University of Cagliari, Cagliari, Italy; 7 The Marcus Neuroscience Institute, Baptist Health South Florida, Boca Raton, USA; 8 Hoffer Consulting, Cleveland, OH 44124 USA; 9 AevisBio, Inc., Gaithersburg, MD 20878 USA

**Keywords:** Neuroinflammation, Immunomodulatory imide drugs (IMiDs), Neurodegeneration, Microglia, Teratogenicity, Pomalidomide, Cereblon, Spalt like transcription factor 4 (SALL4), Traumatic brain injury

## Abstract

**Background:**

Traumatic Brain Injury (TBI) is a major risk factor for neurodegenerative disorders such as Parkinson’s disease (PD) and Alzheimer’s disease (AD), with neuroinflammation playing a critical role in the secondary cell death that exacerbates the initial injury. While targeting neuroinflammation holds significant therapeutic promise, clinical trials of available anti-inflammatory agents have fallen short. 3-Mono-thiopomalidomide (3-MP), a novel immunomodulatory imide drug (IMiD), was designed to curb inflammation without the adverse effects of traditional IMiDs and was evaluated across models involving neuroinflammation.

**Methods:**

3-MP anti-inflammatory activity was evaluated across cellular (RAW 264.7, IMG cells) and mouse studies following lipopolysaccharide (LPS)-challenge (for pro- and anti-inflammatory cytokines/chemokines), and mice subjected to controlled cortical impact (CCI) moderate traumatic brain injury (TBI). 3-MP human cereblon binding, including neosubstrate and molecular modeling evaluation, as well as chicken teratogenicity, ex vivo mouse and human stability studies, and mouse pharmacokinetics were appraised.

**Results:**

3-MP binds human cereblon, a key protein in the E3 ubiquitin ligase complex, without triggering downstream cascades leading to thalidomide-like teratogenicity in chicken embryos. 3-MP reduces pro-inflammatory markers in LPS-stimulated mouse macrophage and microglial cell cultures, and lowers pro-inflammatory cytokine/chemokine levels in plasma and brain of mice challenged with systemic LPS without lowering anti-inflammatory IL-10. 3-MP readily enters brain following systemic administration, and achieves a brain/plasma concentration ratio of 0.44–0.47. 3-MP mitigates behavioral impairments and reduces activation of astrocytes and microglia in mice challenged with CCI TBI.

**Conclusion:**

3-MP represents a promising new class of thalidomide-like IMiDs with potent anti-inflammatory effects that offers potential for treating TBI and possibly other neurodegenerative diseases possessing a prominent neuroinflammatory component.

**Supplementary Information:**

The online version contains supplementary material available at 10.1186/s12929-025-01150-w.

## Introduction

Traumatic brain injury (TBI) is a leading cause of death and long-term disability. Worldwide, an estimated 64 to 74 million people suffer a TBI event annually [1]. In 2020 TBI was considered to have comprised the third largest portion of global diseases [2]. The large majority of TBIs are mild to moderate in character and account for 80–95% of instances, with severe TBI encompassing the balance [3]. With improvements in survival rate following an initial injury, TBI can result in substantial and enduring quality of life detriments that include cognitive, physical, and behavioral impairments that translate into costly long-term access to health care and disability services [3, 4]. TBI can, additionally, considerably increase the risk of later stage neurological [5, 6] and neuropsychiatric disorders [7]. Even with a normalization of neuropsychological and functional measures within a year of a TBI, a half of victims report three or more remaining post-trauma symptoms [8]. TBI is hence considered a progressive disorder, and emerging epidemiological studies suggest that the time-dependent processes triggered by injury can lead to neurodegenerative disorders that include Parkinson’s disease (PD) and Alzheimer dementia [5, 6, 9–12]. Indeed, although conventionally considered an acute brain injury, TBI manifests as a neurodegenerative disorder during its progressive/chronic course [9–13]. Taking these considerations into account, elucidating the mechanism leading to neuronal injury and dysfunction has become vital to the successful development of potentially effective treatment options [13].

Of the two primary phases of TBI-associated brain damage [14], with the initial phase occurring at the time of insult and including contusion, laceration, diffuse axonal injury and intracranial hemorrhage that results in immediate cell death [10, 14, 15], the secondary phase that follows this is widely considered reversible and is hence the major target for TBI therapy. This secondary phase comprises biological cascades initiated at the moment of injury that may persist for much longer, consequent to neuroinflammation, ischemia, glutamate toxicity, astrocyte reactivity, and apoptosis [14, 16–19]. To design rational treatment options, it is imperative to understand the biological processes that drive this delayed TBI secondary phase [10, 13], and reduce them without detrimentally affecting regenerative processes.

Inflammatory cytokines and chemokines appear to be critical to the pathophysiology of TBI as well as to the healing process. Initiation of an inflammatory response is essential for neuro-reparative mechanisms following TBI [19, 20]. However, if excessive or unregulated, these same processes can drive neuronal dysfunction and degeneration by inducing a self-sustaining pathological inflammatory cascade [20, 21]. After TBI, a dramatic and rapid elevation in synthesis and release of pro-inflammatory cytokines ensues from astrocytes and microglia, particularly of tumor necrosis factor-α (TNF-α). Intracerebral TNF-α mRNA and protein levels substantially rise within as little as 17 min after injury in patients dying shortly after a TBI [22]. An alike prompt escalation has been described in rodent TBI models, and a sharp TNF-α increase triggers the elevated generation of multiple other inflammatory cytokines [23–25].

Depending on the engaged signaling pathway, TNF-α can exacerbate oxidative stress, intensify glutamate release, and contribute to blood–brain barrier dysfunction, each of which can aggravate neuronal dysfunction and lead to neuron death [20, 21]. Multiple studies, including our own [23, 26], indicate that neuroinflammation is a central hallmark of TBI. Early neuronal loss provoked by TBI can lead to an extreme neuroinflammatory response that can give rise to ensuing delayed cellular damage. This over-excessive initial TNF-α rise offers a promising drug target that can be mitigated by the immunomodulatory imide drug (IMiD) class that can reduce TNF-α synthesis at both a transcriptional and post-translational level [27].

Pomalidomide (Pom) is considered the most potent of the clinically available IMiDs [28] and, like its analog thalidomide, possesses a core isoindolinone-2-yl-glutarimide structure [27–30]. A concern in relation to repositioning Pom as a TBI treatment is its potential to induce teratogenicity in childbearing women [31, 32]. Although Pom is effective and widely used in treating multiple myeloma that most commonly occurs in those over 60 years age, TBI risk is high among adolescents, young adults, in addition to persons older than 75 years old [2, 3]; thus an IMiD that retains potent anti-inflammatory actions but lacks embryo-fetal toxicity would be desirable [27]. We recently generated and evaluated a novel IMiD, 3,6′-dithiopomalidomide (3,6′-DTP), with such properties that proved more potent than Pom in animal models of TBI and Alzheimer’s disease (AD) [33–37]. Like Pom and thalidomide, 3,6′-DTP potently binds to the E3 ubiquitin ligase targeting protein, cereblon, but does not, however, modify cereblon’s subsequent action to then bind and degrade the key protein SALL4 [35, 37] implicated in classical IMiD teratogenicity [27, 37–42]. Unfortunately, 3,6′-DTP is insufficiently stable to support its use as an oral drug. However, our ex vivo and animal drug metabolism studies identified 3-monothiopomalidomide (3-MP) as a metabolite of 3,6′-DTP and hence a potential clinical drug candidate. Our current studies demonstrate that 3-MP has a long in vivo half-life, is orally bioavailable, enters brain and potently lowers TNF-α generation, binds to human cereblon but does not trigger SALL4 degradation in a manner similar to Pom. 3-MP lacks toxicity in chicken embryos, which is an initial classical model of teratogenicity evaluation. Hence, 3-MP was evaluated for its ability to mitigate secondary TBI phase damage in mice subjected to controlled cortical impact (CCI) injury.

## Methods

### 3-monothiopomalidomide (3-MP)

**Fig. 1:**
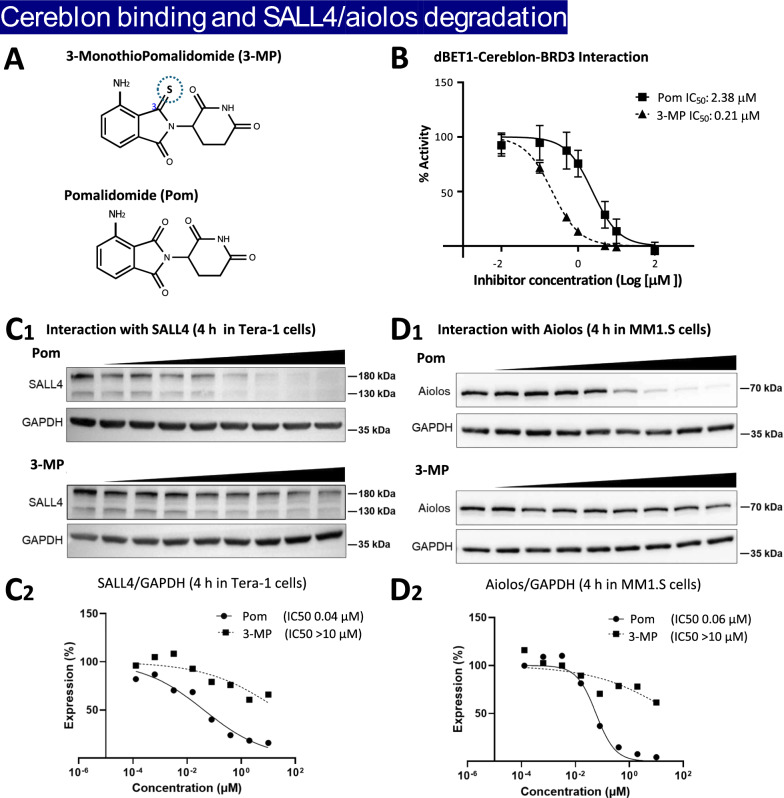
3-MP potently binds to human cereblon but does not trigger degradation of the neosubstrates SALL4 and Aiolos. **A** Chemical structures of 3-monothioPomalidomide (3-MP) and Pomalidomide (Pom). **B** Concentration-dependent evaluation of the interaction between Immunomodulatory drugs (IMiDs) and cereblon by FRET assay provided an IC_50_ value for 3-MP of 0.21 μM and for Pom of 2.38 μM. The thalidomide analog-mediated degradation of neosubstrates, SALL (**C**_**1**_–**C**_**2**_) and Aiolos (**D**_**1**_–**D**_**2**_) were evaluated by Western blotting in Tera-1 cells, and the relative expression level of each neosubstrate was quantified in relation to the housekeeping protein GAPDH (whose level was unchanged by drug incubation). IC_50_ values to lower SALL4 and Aiolos protein expression values were > 10 μM for 3-MP, and 0.04 μM and 0.06 μM for Pom, respectively

3-MP (Fig. 1A) was independently synthesized and chemically characterized by AevisBio (Daejeon, Republic of Korea) and by WL (NIA, NIH, Baltimore, MD, USA). In the present study, 3-MP was generated and provided by AevisBio, which was then independently characterized by WL (Supplemental Information: chemical characterization).

Like other IMiDs, 3-MP is poorly aqueous soluble and, hence, was made up into DMSO for cell culture, and was 'nano-formulated' for animal studies. Specifically, in cellular studies, 3-MP was solubilized in a minimal quantity of DMSO (Sigma, St Louis, MO, USA; Cat # D2650) to which cell culture media was added to achieve the desired concentrations. For animal studies, 3-MP was prepared as a nanosuspension, in line with Cardia et al., 2022 [43]. Specifically, 3-MP was mixed in an aqueous solution (Tween 80/water) and vortexed for 10 min. This coarse drug suspension was transferred into Eppendorf microtubes containing approximately 0.4 g of 0.1–0.2 mm yttrium-stabilized zirconia-silica beads (Silibeads^®^ Typ ZY Sigmund Lindner, Germany). The drug was vortexed twice, at 3000 rpm for 45 min, using a beads-milling cell disruptor (Disruptor Genie^®^, Scientific Industries, USA). The resulting nanosuspension was collected from each microtube and sieved through a 40 µm filter (Cell Strainer, Falcon # 35234) to remove the zirconia-silica beads. Effectively, 87.6 mg of 3-MP was prepared in Tween-80 (60 µl) and made up to a final volume of 8 ml with water (87.6 mg/60 µl/7853µl). The stock drug concentration was 10.83 mg/ml (37.4 mM). The drug vehicle was essentially the same ratio of Tween 80 and water, processed in the identical manner as the drug preparation. 3-MP concentration and stability within the nanosuspension was validated immediately prior to use by LC/MS.

### Cellular culture studies

RAW 264.7 cell culture: RAW 264.7 cells were obtained from ATCC (Manassas, VA, USA) and grown in Dulbecco’s modified Eagle’s (DMEM), high glucose, GlutaMAX™ media supplemented with 10% FBS (ThermoFisher Scientific, Asheville, NC, USA). Cells were maintained at 37 °C and 5% CO_2_, were propagated as described by ATCC guidelines, and were cultured as previously communicated [44]. RAW 264.7 cells were treated either with drug vehicle (DMSO, Sigma, St Louis, MO, USA) or 3-MP (600 nM to 60 μM), with 3–4 wells per treatment group (n = 3–4). One hour later, cells were challenged with lipopolysaccharide (LPS) (20 ng/ml, E. Coli O55:B5, Sigma, St Louis, MO, USA; Cat # L4005). This LPS concentration was selected from pilot studies to provide a submaximal inflammatory insult. Twenty-four hours later, culture media was collected to quantify cell viability (CellTiter 96^®^ Aqueous One Solution Cell Proliferation Assay kit (MTS); Promega, Madison, WI, USA, Cat # G3580), secreted TNF-α protein (ELISA MAX™ Deluxe Set Mouse TNF-α, BioLegend, CA, USA; Cat # 430915) and nitrite (Griess Reagent System, Promega, Madison, WI, USA; Cat # G2930).

IMG cell culture: Mouse immortalized microglial ‘IMG’ cells [45] (Sigma-Aldrich; St. Louis, MO, USA; Cat # SCC-134, RRID:CVCL HC49) were cultured in High Glucose DMEM (Sigma Cat. No. D6546) with 10% heat-inactivated fetal bovine serum (Gibco™ Cat # 10082147), 1 × L-Glutamine (Sigma Cat # TMS-002-C) and 100 U/mL penicillin/streptomycin (Gibco™ Cat # 15140148). IMG cell subculturing was performed according to the product datasheet. In brief, cells were detached from culture plates with Accutase^®^ (Sigma, Cat # A6964), collected, and centrifuged, with the cell pellet subsequently resuspended in media. The cells were then plated for experiments at specified densities and used for a maximum of 10 passages.

For cytokine/nitrite assessment studies, cells were grown in 24-well (40 K cells/well) tissue culture-treated plates in 500 μl of media. The following day, the cells were treated with 3-MP prepared in the same cell culture media (vehicle), at 600 nM to 100 μM. At 24 h, media was collected and replaced with 500 μl fresh media to allow assessment of cell viability (CellTiter 96^®^ Aqueous One Solution Cell Proliferation Assay kit (MTS) (Promega, Madison, WI, USA, Cat # G3580). This colorimetric assay provides a measure of mitochondrial activity, by the conversion of a tetrazolium salt (MTS) into a water-soluble formazan dye by viable cells. 3-MP reduced IMG cell viability at 100 μM (vs. the vehicle control), and hence 3-MP 60 µM was defined as the maximal concentration used in subsequent LPS-induced inflammation-related studies. In these studies, IMG cells were pretreated with vehicle or 3-MP 600 nM to 60 μM and 1 h later, challenged with LPS (10 ng/mL) in the continued presence of drug. This LPS concentration induced a submaximal inflammatory response in IMG pilot studies. Following a 24 h incubation, conditioned media was collected and replaced with 500 μl fresh media to allow assessment of cell viability, TNF-α (ELISA MAX™ Deluxe Set Mouse TNF-α, BioLegend, CA, USA) and nitrite levels (Griess Reagent System, Promega, Madison, WI, USA), according to manufacturer’s instructions.

### Cereblon binding and SALL4/Aiolos degradation

A bead-based AlphaScreen technology was employed to evaluate cereblon binding with nominal changes from the manufacturer’s protocol (BPS Bioscience; Cat # 79770). 3-MP or Pom (as a positive control) were incubated with reaction mixtures that included cereblon/DNA damage-binding protein 1-Cullin 4a-ring-box protein 1 complex (CRBN/DDB1–CUL4 A–Rbx1, 12.5 ng) and bromodomain-containing protein 3 (BRD3) (6.25 ng) in an Optiplate 384-well plate (PerkinElmer; Cat # 6007290). Following 30 min incubation with shaking at room temperature, AlphaLISA anti-FLAG Acceptor and Alpha Glutathione Donor beads (PerkinElmer) were sequentially added and then incubated for a further 1 h at room temperature for each of the added chemicals. Alpha counts were, thereafter, recorded on a Synergy Neo2 (BioTek, Agilent, Santa Clara, CA). The relative activity of the alpha signal was calculated after subtraction of the “blank value” from all readings, and the value of vehicle group was then set as 100%.

The effect of 3-MP activity on SALL4 was evaluated in human Tera-1 cells (an epithelial-like cell originally from a germ-cell tumor of the human testis). This cell line was obtained from the Korean Cell Line Bank (catalog no. 30105; Seoul, Korea), grown in DMEM (supplemented with 10% FBS, penicillin 100 U/mL and streptomycin 100 μg/mL, and maintained at 37 °C and 5% CO_2_). Tera-1 cells were treated with either Pom or 3-MP (0.1 to 10 μM) for 4 h, and their cell lysates were prepared for Western blot analysis, as described previously [33]. Activity of 3-MP and Pom on Aiolos was evaluated in MM.1S (human myeloma) cells (ATCC, Manassas, VA, USA). These were grown in RPMI media supplemented with 10% FBS, penicillin 100 U/mL, and streptomycin 100 mg/mL, and maintained at 37 °C and 5% CO_2_. MM.1S cells were treated with drug (0.1 to 10 μM) for 4 h, and their cell lysates were prepared for Western blot analysis, as described previously [33].

For Western blot analysis, total proteins were extracted using RIPA buffer (Thermo Fisher Scientific, Waltham, MA) containing Halt Protease Inhibitor Cocktail (Thermo Fisher Scientific). Thereafter, proteins were separated by gel electrophoresis and then transferred to polyvinylidene difluoride (PVDF) membranes (Thermo Fisher Scientific), as detailed previously [33, 35]. The following primary antibodies were used: (i) anti-SALL4 antibody (catalog no. ab29112; 1:1000; Abcam, UK); (ii) anti-Aiolos antibody (catalog no. 15103; 1:1000; Cell Signaling Technology, Danvers, MA, USA); and (iii) anti-GAPDH antibody (catalog no. ab8245; 1:5000; Abcam, Cambridge, UK). Subsequent to incubation at 4 °C overnight, the HRP-conjugated secondary goat anti-mouse IgG (Thermo Fisher Scientific) was used to visualize SALL4, Aiolos and GAPDH. GAPDH, a protein that is generally expressed across all eukaryotic cells, was used as an internal control to evaluate SALL4 and Aiolos protein expression levels. Antigen–antibody complexes were detected using enhanced chemiluminescence (Thermo Fisher Scientific, iBright CL1500). Appropriate control studies were undertaken involving the omission of the primary antibody.

### Ex-vivo stability in plasma

An ex vivo evaluation of drug stability was conducted in mouse and human plasma samples (WuXi AppTec, Shanghai, China). 3-MP (10 μL) was added to mouse or human plasma samples (90 μL) to provide a concentration of 100 μg/mL. Samples were, thereafter, incubated at room temperature for 0, 15, 30, 60, 120, and 180 min before immediate drug extraction. Specifically, 3-MP was prepared at a concentration of 1 mg/mL in DMSO and was then appropriately diluted for spiking. Extraction was performed by adding ethyl acetate containing the internal standard (donepezil—known to undergo first-pass hepatic metabolism) to the spiked sample (100 μL), vortexed for 1 min, and centrifuged at 13,000 rpm, 4 °C for 10 min. The supernatant was used for LC–MS/MS analysis. Each sample was analyzed in triplicate utilizing a 12-point calibration curve (0.25, 0.5, 1.0, 2.0, 3.9, 7.8, 15.5, 31.25, 62.5, 125, 250, 500 μg/mL).

LC–MS/MS analysis was performed with a Vanquish Flex UHPLC (Thermo Scientific) and a Orbitrap Exploris 240 mass spectrometer (Thermo Scientific). Samples were separated on a C18 UHPLC column (100 mm × 2.1 mm, 3 μm; Thermo Scientific). The mobile phase A for LC separation was 0.1% formic acid and the mobile phase B was 0.1% formic acid in methanol. The chromatography gradient was designed for a linear increase from 5% B in 1 min, 5% B to 95% B in 8 min, 95% B in 3 min, and 5% B in 3 min. The flow rate was maintained at 0.3 mL/min. Mass spectra were acquired using the data-dependent Top 4 MS2 (ddMS2) method with a mass scan (100–1000 m/z) and 120,000 MS resolution.

### Studies in mice

All mice (Jackson Laboratory, Bar Harbor, Maine, USA) were maintained at 25 °C in a 12/12 h light/dark cycle with access to food and water ad libitum. The experiments conducted in this paper were approved by the Institutional Animal Care and Use Committee of the Intramural Research Program, National Institute on Aging, NIH (animal protocols 331-TGB-2024; 488-TGB-2025). In the current “first in mouse 3-MP study”, experiments were undertaken in male mice only to evaluate whether the drug demonstrated a sufficient signal of efficacy to warrant studies in both sexes, and thereby align with the principles of the “three R’s” in animal research [46]. All experiments were performed during the light cycle portion of the day following the ARRIVE guidelines and recommendations, and all efforts were undertaken to minimize any potential animal suffering and the number of animals used. The latter was achieved by incorporating the outcome measures from our prior studies [14, 33, 35] and performing a power analysis [47].

### Systemic and brain LPS anti-inflammatory studies

8-week-old male C57/BL6 J mice (25–30 g; n = 28) were randomly assigned to five experimental groups. They were administered, via the intraperitoneal (I.P.) route, nano-formulated 3-MP at a dose of 26.5 and 53 mg/kg (equimolar to a clinically translatable dose of 25 and 50 mg/kg thalidomide,) vehicle or Rolipram (10 mg/kg). Rolipram, a discontinued PDE4 inhibitor, with known anti-inflammatory activity [48] was used as a positive control. One hour later, animals were administered either LPS (3 mg/kg, serotype 0111:B4, Sigma Aldrich, in normal saline, 0.1 mL/10 g) or an alike volume of saline (without LPS) via the I.P. route. The LPS dose was selected from prior pilot studies to provide a substantial but sub-maximal rise in plasma pro-inflammatory cytokine levels, in line with our prior research [33, 35, 44]. The selected 3-MP doses were chosen as they are equimolar to, or are less than, doses of thalidomide and analogs that have been verified to be well tolerated in our previous studies [33, 35], and are of translational relevance to humans. Four hours after LPS administration, animals were euthanized, and blood and brain tissue were collected and placed on wet ice. Plasma was quickly separated from blood by centrifugation (10,000*g*, 5 min, 4 °C), and together with a brain sample (cerebral cortex), were stored at − 80 °C. At a later time, cerebral cortex samples were sonicated in a TRIS-based lysis buffer (Mesoscale Discovery) with 3 × protease/phosphatase inhibitors (Halt™ Protease and Phosphatase Inhibitor Cocktail, Thermo Fisher Scientific, Waltham, MA, USA). Samples were then centrifuged (10,000*g*, 10 min, 4 °C) and protein concentrations were quantified by bicinchoninic acid assay (BCA, Thermo Fisher Scientific). Mouse plasma and cerebral cortex samples were analyzed by multi-pro-inflammatory cytokine ELISA (V-PLEX Pro-inflammatory Panel 1 Mouse Kit, Mesoscale Discovery) following the manufacturer’s protocol.

### Pharmacokinetic studies

8-week-old male C57/BL6 J mice (25–30 g; n = 3 to 4 per group) were administered either vehicle or nanoformulated 3-MP at a dose of 2.65 or 26.47 mg/kg by the I.P. route. Mice were euthanized from 30 min to 18 h after dosing. Blood samples were collected into heparinized tubes, centrifuged (10,000*g* × 2 min at 4 °C), and plasma collected and immediately stored at − 80 °C. The brain was rapidly excised on wet ice; a sample of cerebral hemisphere was then removed and immediately frozen to − 80 °C.

### TBI (controlled cortical impact) studies:

TBI studies were, likewise, conducted in 8-weeks-old male C57/BL6 J mice (25–30 g; n = 28) Mice were randomly assigned to four groups prior to surgery: (1) Veh + Sham (i.e., without CCI TBI); (2) Veh + CCI; (3) 3-MP (Low dose (LD), 13.23 mg/kg, prepared in Nano suspension) + CCI; or (4) 3-MP (High dose (HD), 26.47 mg/kg, prepared in Nano suspension) + CCI. All groups were evaluated for the effects of treatment (3-MP or Veh) on CCI, and treatment was administered by the I.P. route (volume: 0.1 ml/10 g body weight) at 30 min and 24 h following CCI or sham challenge. For behavioral analyses, mice were assessed for spatial memory, gait function, motor coordination/balance function and home cage activity. The animals were subsequently evaluated for cellular changes using histology and immunohistochemistry after euthanasia.

In order to develop a controlled cortical impact (CCI) model of TBI, mice were anesthetized with 2.5% 2,2,2-tribromoethanol (Avertin, 400 mg/kg, I.P.) (Sigma, St. Louis, MO, USA) and placed in a stereotaxic frame (Kopf Instruments, Tujunga, CA, USA). A 4 mm craniotomy was performed under sterile procedures; the craniotomy point was selected midway between the lambda and bregma sutures, and laterally midway between the central suture and the temporalis muscle. The skull fragment was then carefully removed without disruption of the underlying dura on the right side of the head. Before injury, the tip of the impactor was angled and kept perpendicular to the exposed cortical surface. The mouse CCI instrument consisted of an electromagnetic impactor, Impact One (Leica Biosystems Inc., Buffalo Grove, IL, USA) that allows independent manipulation of injury severity by adjusting the contact duration and velocity of a metal impactor and the depth of cortical deformation. In these experiments, the contact velocity was set at 5.0 m/s, the dwell time was set at 0.2 s, and the deformation depth was set at 1.5 mm to produce a moderate TBI. The injury site was cleaned and dried by use of sterile cotton tipped applicators prior to suturing the wound. During surgery and recovery, the body temperature of all animals was maintained at 36–37 °C by using a heating pad.

### Behavioral assessment for motor function and coordination

Behavioral tests were conducted 1 week and 2 weeks post the CCI/sham procedure, to evaluate motor function and coordination differences between groups, and to compare with data performed 1 week prior to either procedure. All behavioral tests were conducted during the animals’ day light cycle; home cages were moved to behavioral rooms at least 1 h before testing.

*Home cage activity monitoring* was automatically and non-intrusively monitored using the digital individually ventilated cage system (DVC™; Tecniplast) [49]. Mice were individually housed, and their activity was tracked for 3 days before CCI/sham and 7 days after CCI/sham procedures.

*Beam Walking Test (BWT)* was used to evaluate CCI-induced impairments in motor coordination. Mice have an intrinsic preference for a darkened enclosed environment, as compared to an open bright field. Each mouse was placed in a darkened one-side-open cube box for 2 min for habituation and then was brought to the other (light) end of the beam, to start the trial. Two beams were used in this study with the following dimensions: 0.6 cm or 1.2 cm (width) × 91 cm (length). The time taken for each mouse to traverse the beam to reach the dark cube box and the ‘immobility time’ spent between the moment a mouse was initially placed on the beam and it started walking were recorded. Two trials were recorded for each animal with the 1.2 cm-width beam, and an additional two trials with the 0.6 cm-width were subsequently recorded. The mean times to traverse the beam and the immobility times were calculated, and a plot was generated to evaluate treatment effects; these times were then used for statistical analysis.

*Gait analysis* was employed to assess gait parameters following a specific protocol provided by the DigiGait manufacturer (Mouse Specifics, Inc., Framingham, MA, USA). The study tracked gait function at three time points: baseline, 1-week prior to CCI (PRE), and 1- and 2-weeks post-CCI. At the beginning of the trial, mice were brought to the test chamber for habituation to the new environment for 2 min, and then were given a 1-min run at 5 cm/sec before a 1-min break. The treadmill speed was gradually increased to 15–20 cm/sec for recording, capturing 3–5 s of consistent stepping before returning the animal to its home cage. Videos were assessed using DigiGait software to evaluate parameters that included stride duration, length, paw angle, and stance for all four limbs, following Mouse Specifics, Inc.'s procedures. The parameters measured included brake time (duration from initial paw contact to maximum paw contact after the swing phase), percent of brake phase (percentage of the entire stride duration in the braking phase), propel time (duration from maximum paw contact to just before the swing phase), percent of propel phase (percentage of the total stride duration in the propulsion phase), and paw angle variability (standard deviation of paw angle for the recorded strides). An investigator, blinded to the treatment group, conducted all assessments.

*Asymmetrical motor function* was assessed using the elevated body swing test (EBST), a method initially described by Borlongan and colleagues [50–52]. This test involves suspending animals 10 cm above a testing table and observing their lateral movement or turning while being held by the base of their tail. A swing to the left or right was counted when the animal's head/torso moved more than a 10-degree angle from its vertical axis, after being elevated. The frequency of these swings was measured across 20 consecutive trials. An uninjured animal typically displays an equal frequency of swings to both the left and right sides. The number of contralateral rotations was calculated to generate a mean number of rotations for each treatment group, which was then subjected to statistical analysis.

### Brain tissue processing

Two weeks following injury, mice were deeply anesthetized using isoflurane and underwent transcardial perfusion with 30 ml of phosphate-buffered saline (PBS) followed by 120 ml of 4% paraformaldehyde (PFA) in PBS. The brains were collected and placed into a 4% PFA solution for post-fixation overnight, and then transferred to 25% followed by 30% sucrose solution in PBS for dehydration. Sections of a region extending from the striatum to the hippocampus were cut coronally into 25 μm thickness using a cryostat (Leica Biosystems Inc., Buffalo Grove, IL, USA) and stored in a cryoprotectant solution. These sections were subjected to Giemsa (nuclear/chromosomal) staining and immunohistochemical analysis.

### Quantification of lesion volume and enlargement of ventricle area after TBI

Brain sections obtained 2 weeks post-TBI, with a thickness of 25 µm, were placed onto slides. These sections were stained using a 10% Giemsa KH_2_PO_4_ buffered solution (pH 4.5) for 30 min at 40 °C. Following a rinse, the slides underwent de-staining, differentiation, dehydration in absolute ethanol, cleared in xylene, and then were cover-slipped. Subsequently, the slides were scanned using an All-in-One Fluorescence Microscope BZ-X710 (Keyence Corporation of America, Itasca, IL, USA). Brain image areas were quantified using ImageJ 1.52q software (National Institutes of Health, Bethesda, MD, USA). The contusion volume size and lateral ventricle size were determined as described previously [14, 53], using the calculation formulas as follows:

*Contusion volume size:* Σ (area of contralateral hemisphere–area of ipsilateral hemisphere)/Σ area of contralateral hemisphere × 100.

*Lateral ventricle size:* Σ area of ipsilateral lateral ventricle/Σ area of contralateral lateral ventricle.

Nine brain sections from each mouse were quantified, spanning regions from bregma 0.86 mm to − 1.46 mm.

### Immunofluorescence of GFAP and IBA1 in the cortex

Immunohistochemistry for GFAP and IBA1 was performed as described previously [53]. Briefly, brain sections were incubated with blocking buffer (4% bovine serum albumin (BSA)) for 1 h, followed by overnight incubation in primary antibodies at 4 °C: goat anti-GFAP (Glial Fibrillary Acidic Protein) (1:500; Abcam) or rabbit anti-Iba1 (1:500; FUJIFILM Wako Pure Chemical Corporation). After washing, the sections were treated with secondary antibodies (conjugated with Alexa Flour 488 or 555) for 3 h at room temperature. Subsequently, the sections were washed, mounted with Antifade Mounting Medium with DAPI (Vector Laboratories), and cover-slipped. Using a Confocal Laser Scanning Microscope (Zeiss 710), four images per mouse brain were captured, and cell numbers in each image were counted using ImageJ 1.52q software (NIH). Controls involved (i) omitting the primary antibody (a control to define the nonspecific binding of the secondary antibody); and (ii) observers were blinded to treatment groups. Immunofluorescence analysis targeted Iba1-positive microglia and GFAP-positive astrocytes using a × 40 oil magnification objective. For each mouse, four to six cortex fields from both hemispheres were captured. Both the number and mean intensity of GFAP and IBA1-positive cells in each field were quantified using ImageJ 1.52q. Observers remained blinded to the treatment group across all analyses.

### Morphological analysis of astrocytes in the cortex

Image acquisition: For the 3D reconstruction of astrocytes, Z-stack images (25 μm depth, 1 μm steps, × 40 magnification) of the cortex (both ipsilateral and contralateral) were acquired using a Zeiss LSM 880 confocal microscope (1024 × 1024 pixels, 32-bit depth, pixel size 0.63 μm, zoom 1.0) (see Fig. 7A). Images were captured with consistent settings. No frame averaging or accumulation was performed. Raw.czi files were used for further analysis using IMARIS software (Version 9.31, Oxford Instruments).

Surface reconstruction: IMARIS was utilized to reconstruct the astrocyte surface using the following custom settings: surface detail at 0.46 μm (smooth), background subtraction for thresholding (local contrast), and the diameter of the largest sphere fitting into the object set at 1.70 μm. The color base was set to diffusion transparency of 55%. The filter function was applied to remove non-specific background signals using the volume max–400 μm^3^ setting. Astrocytes with incomplete somata (cut by the x, y, or z plane) and reconstructed entities clearly not representing astrocytes (e.g., filaments without soma) were manually removed. The final surface reconstruction was created using the “mask all” function.

Filament reconstruction: The surface reconstruction served as the template for filament reconstruction with the following custom settings: detect new starting points with a largest diameter of 6.00 μm, seed points at 0.250 μm, and removal of seed points around starting points within a 15 μm sphere diameter. Seed points were manually corrected if incorrectly placed by the IMARIS algorithm. All surface and filament parameters were exported into Excel files for data analysis.

Quantification: Approximately 20–25 cells per animal were analyzed (2 ROIs/ipsilateral and 2 ROIs/contralateral side for one brain slice × 3 brain slices = 6 ROIs/ipsilateral and 6 ROIs/contralateral side × 3–4 cells/ROI = ∼20–25 cells/hemisphere/animal). Each Z-stack reconstruction took approximately 15 min, including manual removal of astrocytes. All images were analyzed blindly with respect to experimental conditions.

Sholl analysis: Sholl analysis [54] was performed in IMARIS using the filament reconstruction mode. Data sets were exported to Excel for further analysis. The total number of Sholl intersections was calculated by summing all intersections from each individual sphere per astrocyte.

### Morphological analysis of microglia in the cortex

In relation to Iba1 immunostaining, microglial cells were categorized into various morphological subtypes, including ramified, intermediate, amoeboid, and round shapes. These subtypes are indicative of different functional states of microglia. To analyze these morphological parameters, MotiQ Software was employed [55], which is a publicly available automated analysis tool developed as an ImageJ plugin in Java. The microglial ramification index, a key parameter, measures the complexity of the cellular shape by comparing the cell's surface area to that of a perfect sphere with the same volume as the cell being analyzed. A ramification index of 1 represents a completely round cell without any processes. Higher values indicate a deviation from a perfectly round shape, with more branches and a more complex 3-dimensional (3D) structure. All image segmentation and quantification were conducted on maximum intensity projections of 3D image data, enabling a comprehensive assessment of microglial morphology.

### Molecular modeling 3-MP docking with human cereblon

Molecular docking studies were performed on 3 different proteins (PDB IDs- 4 TZ4, 6UML and 7BQU) using the Glide (grid-based ligand docking) program incorporated in the Schrödinger molecular modeling package with extra precision (XP). These three human cereblon protein structures were chosen to enhance the thoroughness and reliability of resulting docking predictions through Structural Diversity, Accuracy, Binding Site Variability and Redundancy and Validation. All three PDB entries contain native ligands within their structures, specifically, lenalidomide, pomalidomide and s-thalidomide, respectively. The ‘protein preparation wizard’ within the Schrödinger module was used to build the structures. This involved addition of hydrogen atoms and removal of water molecules beyond 5 Å of the binding sites. The active site grids were generated using the Receptor Grid Generation tool in the Glide module (Maestro, Schrödinger Release 2023–2). In order to execute the docking experiment, re-docking of all the native ligands was performed. LigPrep (Schrödinger) was used to test the native ligands for chiralities and to then transform them into a 3D structure (LigPrep, Schrödinger Release 2023–2). The OPLS_2005 force field was applied to induce ionization and tautomeric states. The Schrödinger molecular modeling package's Glide (grid-based ligand docking with extra precision (XP)) tool was employed to improve molecular docking accuracy. Thereafter, RMSD (Root Mean square Deviation) scores, a metric of how similar two or more protein structures are, were calculated to validate the docking experiments, and 3-MP was evaluated to decipher its docking within human cereblon structures through docking score and associated interactions.

### Chicken embryology effects of 3-MP

All studies with chicken embryos were performed in line with UK Home Office regulations and followed guidelines, standards and practices governed by the University of Aberdeen Ethics Committee (Scotland, UK). Fertilized chicken eggs were acquired from Henry Stewart & Co Ltd, Norfolk UK. Each working solution of 3-MP included DMSO at 1% (doses applied were between 2.65 and 132ug of 3-MP). Embryos were incubated at 37 °C to reach E2.5 (early developmental stages). Eggs were then opened and the embryonic membranes protecting the embryos were removed with forceps. Chicken embryos usually lie on one side, so the left side is directly against the yolk and the right side can be observed. Drug (3-MP) or Control (1% DMSO alone) solutions were applied in 100 μL aliquots over the middle of the embryo on its right side. Embryos were maintained at room temperature for 20 min before being replaced in a 37 °C incubator for evaluation 24 h thereafter. Consequent to limited drug diffusion when applied to the right side of an embryo, the right side is deemed the ‘treatment’ side and the left (facing internally towards the yolk sac) is considered normal after treatment and thus can act as an internal control.

### Statistical analysis

In the analysis of behavioral data, a two-way repeated measure analysis of variance (ANOVA) was employed to evaluate both group and time factors. Subsequent multiple within-subject comparisons were conducted using Dunnett’s test when a significant main effect of time was observed. For the analysis involving pro-inflammatory cytokine ELISAs, contusion volume size, and lateral ventricle size, a one-factor repeated measures ANOVA was used to compare the four groups of data, followed by Dunnett’s test specifically at the two-week post-lesion time point. For the morphological analysis, a two-way repeated measure analysis of variance (ANOVA) was employed. Subsequent multiple within-subject comparisons were conducted using Dunnett’s test (with Bonferroni correction for multiple comparisons, when appropriate). The statistical analysis was performed using GraphPad Prism 7 (San Diego, CA, USA), with a predefined significance level of p < 0.05 for each assessment. All presented data are expressed throughout the text as the mean ± standard error of the mean (SEM). The number of samples per group (n) is provided within each Figure/Figure legend.

## Results

### 3-MP potently binds cereblon but does not lower the expression of the cereblon neosubstrates SALL4 and Aiolos

Prior investigations have established that the principal process underpinning the teratogenic actions of classical IMiDs arises from their binding to human cereblon, the target-associated protein of the E3 ubiquitin ligase complex [27, 37–42]. This modifies the complex’s neosubstrate preference to recognize and catalyze the ubiquitination of SALL4 [37–42]. Human mutations in the *SALL4* gene associate with developmental complications in patients with Okihiro/Duane-Radial-ray syndrome and Holt–Oram syndrome [56–58] that share key features with thalidomide embryopathy [59]. In consideration of this, the binding of 3-MP to human cereblon, together with its potential to reduce the expression of SALL-4 in Tera-1 cells (a human immortalized carcinoma line) were evaluated, using Pom as a positive control. Expression of the protein Aiolos, via which IMiDs are considered to mediate their anticancer actions, were similarly evaluated in MM.1S cells (a human multiple myeloma line).

Potent interaction with human cereblon through a cereblon/BRD3 binding FRET assay was found for both 3-MP and Pom, with IC_50_ binding values of 0.21 and 2.38 µM, respectively (Fig. 1B). Evaluating drug concentrations that skirted this range (0.1 to 10 μM), 3-MP minimally reduced SALL4 protein expression in Tera-1 cells (Fig. 1C) and Aiolos expression in MM.1S cells (Fig. 1D) (IC_50_ values > 10 µM). In contrast, identical incubation with Pom resulted in substantial reductions in SALL4 and Aiolos protein expression levels (IC_50_ values 0.04 and 0.06 µM, respectively).

### Molecular modeling of 3-MP with human cereblon and SALL4

**Fig. 2:**
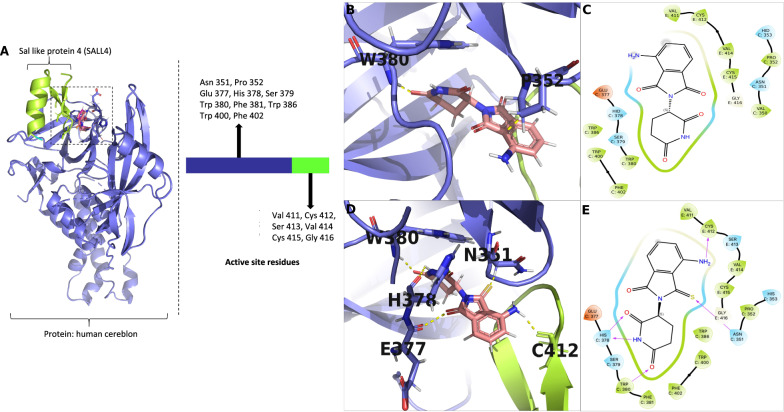
Docking showing the structural basis for thalidomide teratogenicity revealed by the Cereblon-DDB1-SALL4-Pomalidomide complex (PDB ID:6UML) into which 3-MP is docked. **A** 3-D structure of human cereblon (blue) and sal-like protein 4 (SALL4) (green) define the binding site for thalidomide-like compounds and key amino acids involved. **B** 3-D interactions of pomalidomide with interacting amino acids of Cereblon-DDB1-SALL4-Pomalidomide complex; **C** 2-D interactions of pomalidomide with interacting amino acids of Cereblon-DDB1-SALL4-Pomalidomide complex. **D** 3-D interactions of 3-MP with interacting amino acids of Cereblon-DDB1-SALL4-Pomalidomide complex and **E** 2-D interactions of 3-MP with interacting amino acids of Cereblon-DDB1-SALL4-Pomalidomide complex

As an initial measure to assess and validate the utility of the molecular modeling program, docking of co-crystal ligands similar to 3-MP, specifically, the clinically approved IMiDs lenalidomide, pomalidomide, s-thalidomide, was performed to analyze their structural interaction with human cereblon protein PDB IDs 4 TZ4, 6UML and 7BQU. Removal of the native ligand and redocking showed the lowest score − 12.95 kcal/mol for pomalidomide within 6UML, whereas the 4 TZ4-lenalidomide and 7BQU-s-thalidomide complexes generated scores of –10.89 and –10.94 kcal/mol scores, respectively (Supplemental Table S1). In each case the RMSD values were low (< 0.2 Å), providing an indication of close replication of previously reported dockings [40, 60, 61]. A comparison of docking across all PDB entries suggests that the glutarimide moiety common across all the evaluated compounds docked within a hydrophobic pocket formed by H378, S379, W380, F381, W386, W400 and F402 residues (Fig. 2, Supplemental Fig. S1 and S2). On evaluating the docking of 3-MP across the human cereblon PDB IDs 4 TZ4, 6UML and 7BQU, the lowest docking score was − 12.47 kcal/mol (6UML), followed by − 12.17 and − 11.69 kcal/mol (7BQU, 4 TZ4, respectively) (Supplemental Table S2). By far the majority of the key interactions, including hydrogen bonds and polar interactions, were retained (Fig. 2, Supplemental Figs. S1 and S2) and comparison between ligand and pose conformation similarly provided a low RMSD (not shown). Hence, these PDB coordinates can be considered to dock 3-MP in a manner that closely aligns with the core group bonds of the glutarimide ring across all the PDB entries.

Key interactions between the human cereblon-DDB1-SALL4 complex and the bicyclic moiety of 3-MP were achieved via N351, E377 and C412 (Fig. 2), with the latter amino acid interacting with the 3-position thiocarbonyl of the 3-MP. H378 and W380 interacted with the glutarimide ring. Notably, stronger binding interactions for 3-MP due to its structural features were determined as compared to pomalidomide. This aligns with the experimentally measured more potent IC_50_ value of 3-MP to human cereblon (Fig. 1B), but does not explain the similar docking scores in Supplemental Table S1 and S2.

### Chicken embryology effects of 3-MP

Evaluation of 3-MP in early chicken embryos (E2.5) was performed across a range of concentrations (Table 1) from very low to high (2.65 to 132 ug). Chicken embryos, following 24 h of exposure to 3-MP, showed no adverse developmental differences or damage when compared to DMSO controls.

**Table 1:** Dose and actions of 3-MP on chicken embryos at E2.5 developmental stage

3MP Dose (ug)	N	E2.5		
		Normal	Abnormal	Not counted
2.65	2	2	–	–
5.3	3	3	–	–
20	5	5	–	–
26.4	7	5	–	2^a^
52.8	5	3	–	2^a^
66	3	3	–	–
132	3	3	–	–

^a^Embryos died very soon after drug application due to damage incurred when accessing the embryo. Deaths were considered methodology-related rather than drug-induced

Evaluation of chicken embryos was performed at 24 h following dose-dependent 3-MP administration at E2.5 developmental stage (N = 2–7 per group)

### 3-MP mitigates LPS-induced inflammation in RAW 264.7 and IMG cells

For this study, both cultured RAW 264.7 and IMG cells were pre-administered either vehicle or 3-MP (0.6μM to 60μM), and then challenged with LPS at a concentration of 20 ng/mL and 10 ng/mL, respectively, one hour later to induce inflammation. Following this procedure, these mouse immortalized macrophage/microglia cells provide a useful phenotypic model to screen for anti-inflammatory drug action [44, 62, 63], as they share features with brain-derived microglial cells in response to a pro-inflammatory signal. After 24 h of LPS exposure, cellular viability along with levels of nitrite (a stable marker of nitric oxide generation) and TNF-α were quantified in both cell lines.

**Fig. 3:**
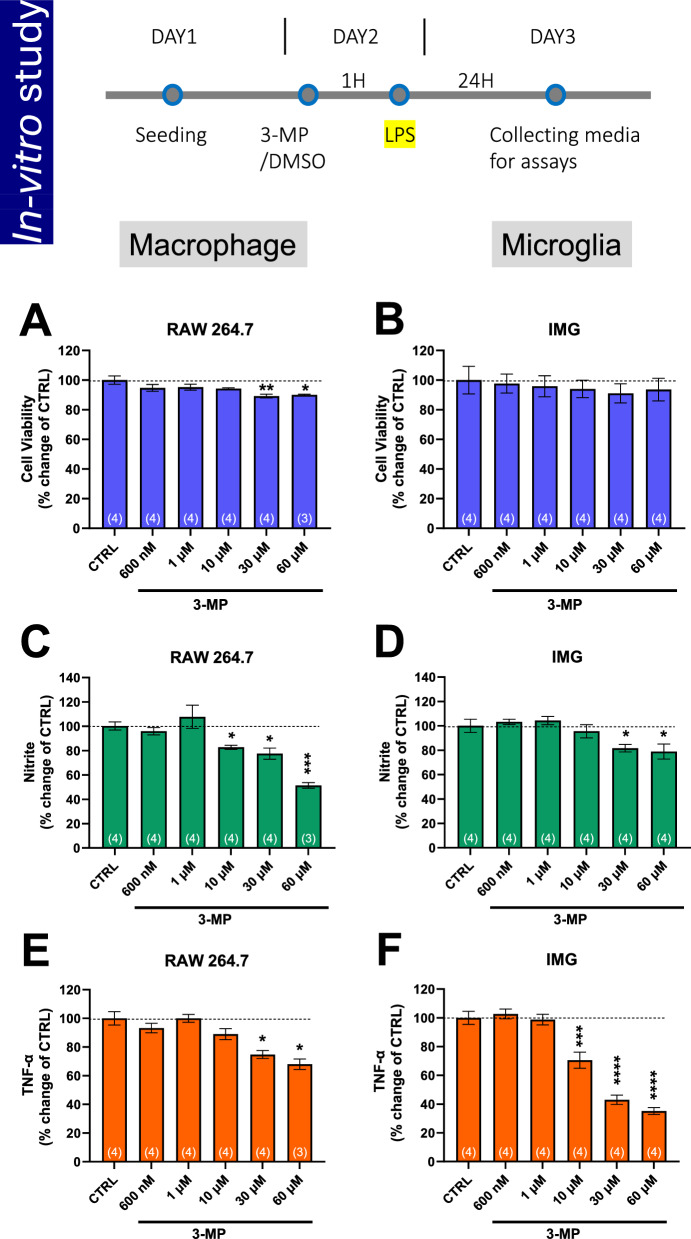
3-MP mitigates LPS-induced inflammation in cultured RAW 264.7 and IMG cells. Cultured RAW 264.7 and IMG cells were pretreated with either vehicle or 3-MP (0.6 − 60 μM) and challenged with LPS (20 ng/mL) 1 h later. At 24 h following LPS challenge, cellular viability (**A**, **B**), nitrite (a stable marker of NO generation) (**C**, **D**), and TNF-α levels (**E**, **F**) were quantified. 3-MP significantly lowered LPS-induced elevations in nitrite and TNF-α levels. *p < 0.05, **p < 0.01, ***p < 0.001, ****p < 0.0001 vs. the control (LPS + Veh) group

3-MP proved well-tolerated across RAW 264.7 and IMG cells, maintaining cellular viability at levels exceeding 90% across all evaluated concentrations (Fig. 3A, B). Notably, 3-MP significantly reduced LPS-induced levels of nitrite (for RAW 264.7 cells: ≥ 10 μM = p < 0.05; for IMG cells: ≥ 30 μM = p < 0.05) (Fig. 3C, D) and TNF-α (for RAW 264.7 cells: ≥ 30 μM = p < 0.05; for IMG cells: ≥ 10 μM = p < 0.001) (Fig. 3E, F); thereby, demonstrating anti-inflammation in cellular studies.

### 3-MP is stable in plasma samples and enters the brain following systemic dosing

**Fig. 4:**
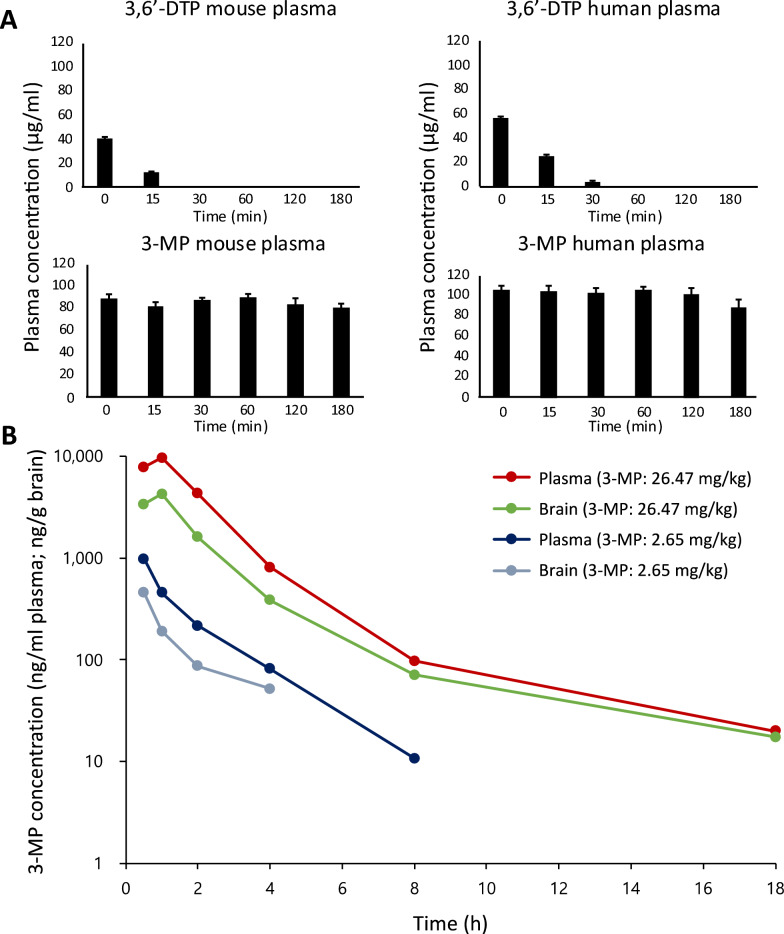
Time-dependent ex vivo and in vivo pharmacokinetics of 3-MP. **A** Ex-vivo disappearance of 3,6′-DTP (100 μg/ml) in mouse (upper left) and human plasma (upper right), and of 3-MP (100 μg/ml) in mouse (lower left) and human plasma (lower right)—expressed as a percent of compound initially added (at time zero) (n = 3). **B** Plasma and brain 3-MP concentrations in mice following I.P. administration of 3-MP high (26.5 mg/kg) and low (2.65 mg/kg) doses (n = 3 to 4 animals per time point). Of note, the data and Figure ((A) lower right panel) relating to the ex vivo stability of 3-MP in human plasma derives from [64]—study evaluating the stability and pharmacokinetics of 3-MP in rat, with a comparison to human

Unlike 3,6′-DTP that rapidly disappears on addition to mouse and human plasma samples, 3-MP proved to be stable in plasma (Fig. 4A), in accord with its stability in rat plasma [64]. In in vivo studies in mouse, Fig. 4B shows the time-dependent plasma and brain drug levels achieved by 3-MP following its I.P. administration at doses of 26.47 mg/kg and 2.65 mg/kg. Concentrations within the plasma and brain compartments largely parallel one another. Pharmacokinetic parameters are provided in Table 2. Notably, amongst these, the brain/plasma drug concentration ratio varied between 0.43 and 0.47 for the two evaluated doses (calculated from the respective AUC_infinity_ values).

**Table 2:** Pharmacokinetic parameters of 3-MP following its I.P. administration to mice

Drug dose	Parameter	Plasma	Brain
3-MP 26.5 mg/kg	C_Max_	9576.00 ng/ml	4227.60 ng/g
	T_Max_	1.0 h	1.0 h
	AUC_Last_	20,718.30 ng.h/ml	8968.50 ng.h/g
	AUC_Inf_	20,775.67 ng.h/ml	9052.48 ng.h/g
	Brain/plasma ratio	0.44	
3-MP 2.65 mg/kg	C_Max_	977.80 ng/ml	453.60 ng/g
	T_Max_	0.5 h	0.5 h
	AUC_Last_	1414.35 ng.h/ml	550.20 ng.h/g
	AUC_Inf_	1435.71 ng.h/ml	676.54 ng.h/g
	Brain/plasma ratio	0.47	

### 3-MP attenuates systemic and brain inflammation following LPS challenge in mice

**Fig. 5:**
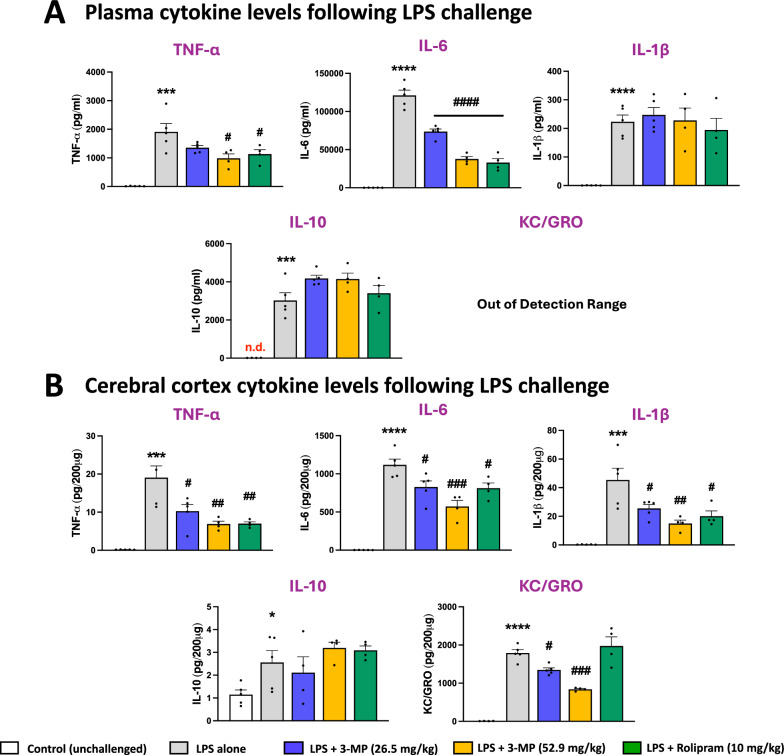
3-MP mitigates LPS-induced elevations in key pro-inflammatory cytokines without altering anti-inflammatory cytokines in plasma (**A**) and cerebral cortex (**B**). Systemic administration of LPS (3 mg/kg, I.P.) to mice induced significant increases in pro- (plasma: TNF-α, IL-6, IL-1β, KC/GRO; brain: TNF-α, IL-6, IL-1β, KC/GRO) and anti-inflammatory (plasma and brain: IL-10) cytokines at 4 h. One hour pre-treatment with 3-MP (26.5 and 53 mg/kg, I.P.) significantly mitigated LPS-induced pro-inflammatory cytokines changes (plasma: TNF-α, IL-6; brain: TNF-α, IL-6, IL-1β, KC/GRO) in the absence of alteration in anti-inflammatory IL-10. *p < 0.05, ***p < 0.001, ****p < 0.0001 refers to the effects of LPS compared to the control value (drug vehicle + saline without LPS). #p < 0.05, ##p < 0.01, ###p < 0.001, ####p < 0.0001 refers to the effect of drug treatments vs. drug vehicle + LPS. Rolipram (10 mg/kg, I.P.), a discontinued PDE4 inhibitor with known anti-inflammatory activity [48], was used as a positive control. Values are presented as mean ± S.E.M., of n observations (Control, n = 5; LPS alone, n = 6; 3-MP (Low) + LPS, n = 5; 3-MP (High) + LPS, n = 4; rolipram + LPS (positive control), n = 4)

In the light of the ability of 3-MP to dose-dependently blunt LPS-induced inflammation in cell culture, as an initial in vivo efficacy evaluation we quantified the ability of 3-MP to mitigate LPS-mediated increases in pro-inflammatory cytokines in mice; this is a classical animal model for anti-inflammatory drug discovery [65]. In line with our previous studies [33–35], systemic LPS administration (3 mg/kg, I.P.) triggered statistically significant increases in key pro- and anti-inflammatory proteins in both plasma and brain, on measurement at 4 h [an approximate steady-state time for plasma TNF-α generation in response to LPS [66]) (Fig. 5. Plasma (A), brain (B)]. Both evaluated doses of 3-MP (26.5 and 53 mg/kg, I.P.) lowered LPS-mediated elevations in plasma TNF-α and IL-6 (p < 0.05 to < 0.0001), with the higher 3-MP dose demonstrating the greatest efficacy (Fig. 5A). A similar action was apparent in brain, where 3-MP-mediated a dose-dependent reduction in TNF-α, IL-6, IL-1β and KC/GRO. (Fig. 5B). Importantly, levels of the anti-inflammatory cytokine IL-10 were unaffected as compared to LPS treatment.

### CCI-induces a contusion and increase in ventricle size that are unimpacted by 3-MP

**Fig. 6:**
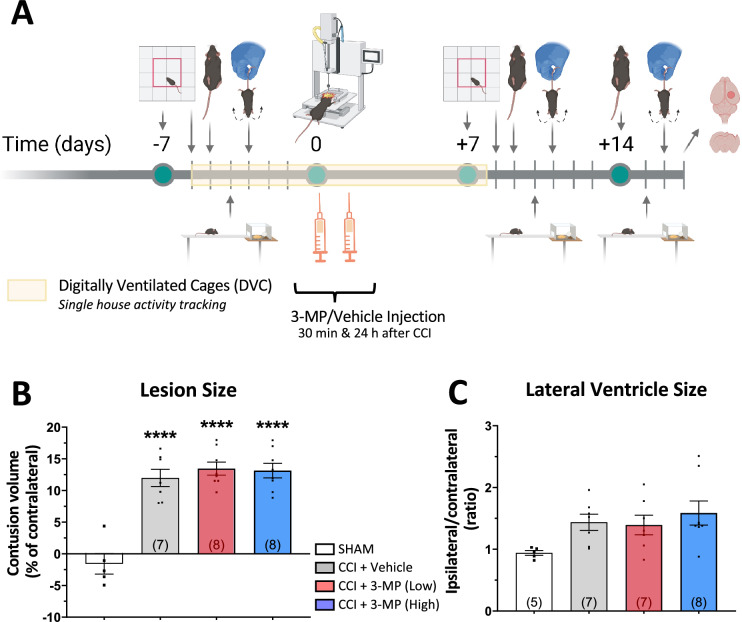
Timeline of mouse model of TBI with 3-MP treatment experimental design and contusion and lateral ventricle size at two weeks. **A** Mice were first evaluated for their baseline novelty exploration memory, gait function, asymmetrical motor function and motor coordination/balance function by novelty-dependent exploration test, DigiGait analysis, elevated body swing test and beam walking test at one week prior to and at 7 and 14 days after CCI or sham injury. Single house activity tracking was conducted from day 6 prior to CCI to day 7 after CCI TBI by Digital Ventilated Cage (DVC) system. Mice received two injections of 3-MP or Vehicle at 30 min and 24 h following CCI TBI. After two weeks, mice were euthanized, followed by perfusion for assessment of histology and immunohistochemistry. Thereafter, **B** TBI contusion volume and **C** lateral ventricle size were quantified from 25 µm Giemsa-stained coronal brain sections (n = 5 to 8 animals/group; ****p < 0.0001 vs. SHAM group w/o CCI TBI)

A direct effect of CCI challenge was a loss of cerebral cortical tissue at the TBI site, which is expressed as a percent of the contralateral hemisphere in Fig. 6B (p < 0.0001 vs. sham). Lateral ventricle enlargement was, likewise, increased by CCI (Fig. 6C). These measures at 2-weeks following CCI were unchanged by 3-MP, indicating alike TBI-induced damage was instigated by CCI across groups.

### 3-MP attenuates CCI-induced astrocyte activation and induces changes in astrocyte morphology

**Fig. 7:**
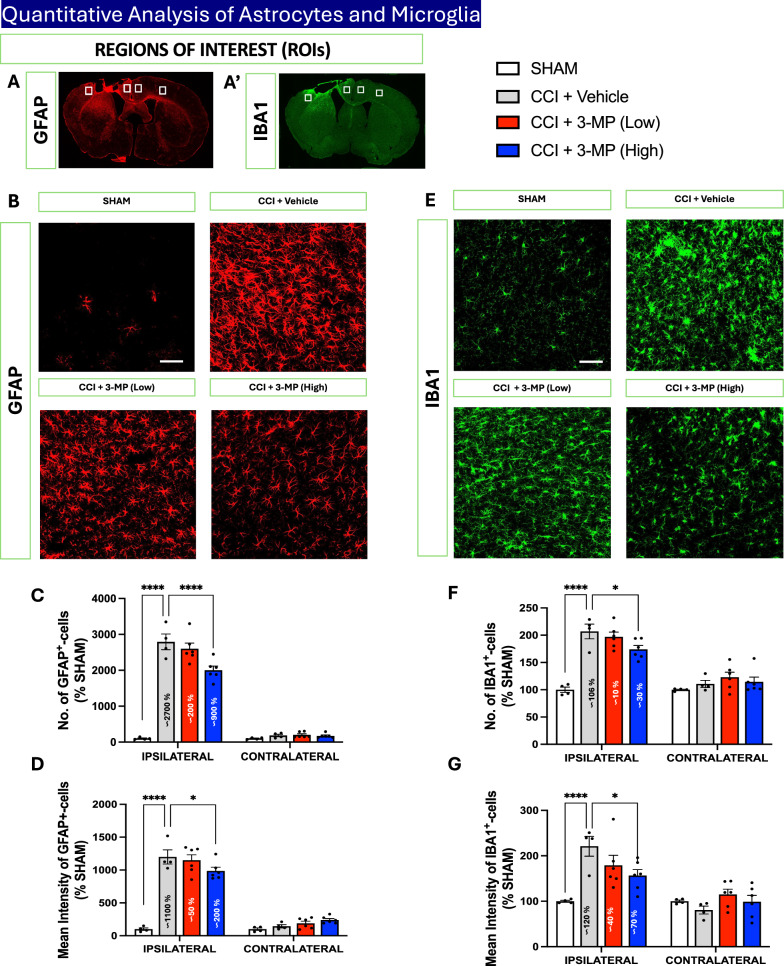
3-MP treatment ameliorates astrocytes and microglia activation following CCI injury. Region of Interests (ROIs) of ipsilateral and contralateral cortex used for the quantitative analysis of GFAP (**A**) and IBA1 (**A**′)-positive cells. Representative images of GFAP-positive cells in the ipsilateral cortex (**B**). Histogram indicates the number (**C**) and mean intensity (**D**) of GFAP-positive cells. Representative images of IBA1-positive cells in the ipsilateral cortex (**E**). Histogram indicates the number (**F**) and mean intensity (**G**) of IBA1-positive cells. Values are expressed as a percentage of the SHAM group (n = 5 to 8 animals/group). Black dots within bars represent the values of individual mice; Two-way ANOVA with Dunnett's multiple comparisons test, error bars representing mean ± SEM; *p < 0.05, ****p < 0.0001, SHAM or CCI + 3-MP (Low) or CCI + 3-MP (High) versus CCI + Vehicle. Scale bar = 50 μm

To investigate the effects of 3-MP on TBI-induced astrocyte activation in the cerebral cortex of mice subjected to CCI, we assessed GFAP reactivity in both the ipsilateral and contralateral cortex. GFAP, an intermediate filament protein, serves as a classical histological marker for reactive astrogliosis, with its activity directly correlated to the severity of, and proximity to, injury in the brain. We observed a significant increase in both the number (p < 0.0001) and mean intensity (p < 0.0001) of GFAP^+^-cells in the ipsilateral cortex of CCI mice compared to the sham controls (Fig. 7A–D). Notably, a high dose of 3-MP (26.47 mg/kg) significantly mitigated TBI-induced astrocyte activation, as evidenced by a substantial decrease in the number (p < 0.0001) and mean intensity (p < 0.05) of GFAP^+^-cells, as compared to the CCI + vehicle group (Fig. 7A–D). In contrast, the low dose of 3-MP (13.23 mg/kg) did not affect TBI-induced changes in astrocyte activation.

**Fig. 8:**
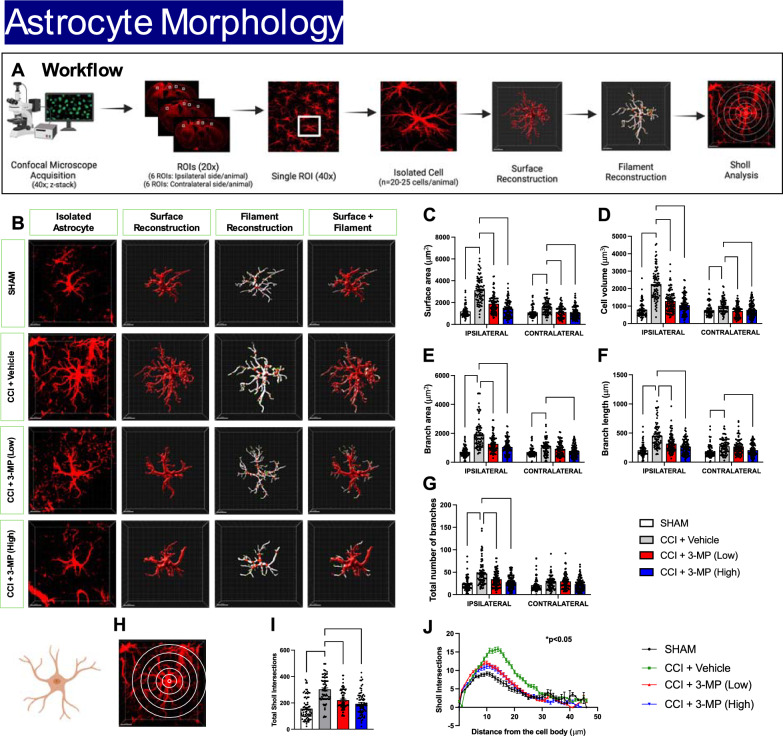
3-MP treatment reduces morphological alterations in astrocytes following CCI injury. The schematic illustrates the workflow for morphological analysis of 3D-reconstructed astrocytes in the ipsilateral and contralateral cortex (**A**). Representative images of 3D-reconstructed astrocytes from ipsilateral cortex (**B**). Histogram indicates the surface area (**C**), cell volume (**D**), branch area (**E**), branch length (**F**), and total number of branches (**G**) of GFAP-positive cells. Sholl analysis of 3D-reconstructed astrocytes (**H**–**J**). Representative image of sholl radii on isolated 3D-reconstructed astrocyte (**H**). Histogram indicates the total sholl intersection (**I**), and mean distribution plot of the number of Sholl intersections as a function of the distance from the cell body (**J**). Black dots within bars represents values from individual 3D-reconstructed astrocytes (n = 20–25 cells/animal) (n = 5 to 8 animals/group); Two-way ANOVA with Dunnett's multiple comparisons test, error bars representing mean ± SEM; *p < 0.05, **p < 0.01, ***p < 0.001, ****p < 0.0001, SHAM or CCI + 3-MP (Low) or CCI + 3-MP (High) versus CCI + Vehicle. Scale bar = 50 μm

Morphological analysis: To further elucidate morphological changes in astrocytes following TBI and the potential modulatory effects of 3-MP, we conducted a comprehensive morphological analysis of astrocytes in the cortex using IMARIS software. We acquired 25 μm z-stack confocal images (32-bit, 1 μm steps, × 40 objective) of brain slices stained with GFAP and exported.czi files for further analysis. Astrocytes with incomplete staining were excluded from the analysis (for detailed IMARIS-based analysis, refer to the “Materials and Methods” section). This methodology enabled unbiased high-throughput analysis of various cellular features including surface area, cell volume, branch area, branch length, and total number of branches (Fig. 8A). Mice subjected to CCI demonstrated significant alteration in these morphological features compared to sham controls (for all parameters: p < 0.0001) (Fig. 8B–G). Importantly, both the low and high doses of 3-MP significantly ameliorated TBI-induced morphological alterations in astrocytes in both the ipsilateral (for all parameters: p < 0.0001) and contralateral cortex (Fig. 8B–G).

Sholl analysis: To assess the complexity and ramification of astrocytes, we performed a Sholl analysis on individually 3D-reconstructed astrocytes. Pro-inflammatory astrocytes are known to undergo extensive ramification, characterized by increased process extension and complexity, and release of pro-inflammatory cytokines. To this end, we superimposed spheres of increasing radius (1 μm increase in radius per step, Fig. 8H) starting at the center of the soma, and then measured the number of process intersections that each sphere encountered. Astrocytes from the CCI + vehicle group displayed a significant increase in complexity, indicated by a higher number of total Sholl intersections, as compared to astrocytes from the sham group (p < 0.0001). This increased complexity was significantly mitigated by both low and high doses of 3-MP (p < 0.0001) (Fig. 8I, J).

### 3-MP mitigates CCI-induced microglial activation and microglial morphology

Next, to evaluate the effects of 3-MP on CCI-induced microglial activation, we examined IBA1 immunoreactivity in both the ipsilateral and contralateral cortex (Fig. 7). IBA1 is a well-established marker for microglial activation, with its expression upregulated in activated microglia, making it a valuable indicator of microglial response to injury [67, 68]. We observed a significant increase in both the number (p < 0.0001) and mean intensity (p < 0.0001) of IBA1^+^-cells in the ipsilateral cortex of CCI mice compared to the sham controls, indicative of ongoing microgliosis (Fig. 7A′, E–G). Notably, high dose 3-MP (26.47 mg/kg) significantly mitigated TBI-induced microgliosis and microglial activation, as evidenced by a substantial decrease in the number (p < 0.05) and mean intensity (p < 0.05) of IBA1 + -cells, as compared to the CCI + vehicle group (Fig. 7A′, E–G). In contrast, low dose 3-MP (13.23 mg/kg) did not significantly affect the TBI-induced microglial activation.

**Fig. 9:**
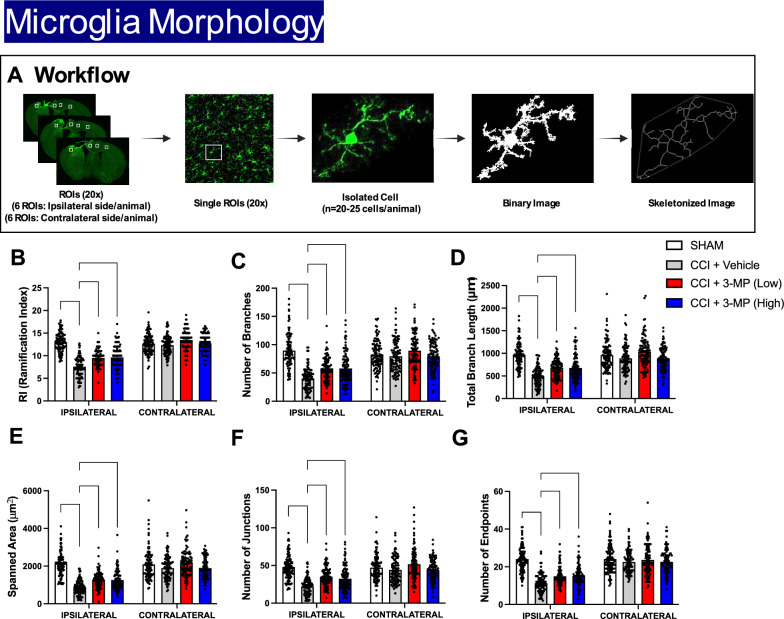
3-MP treatment reduces morphological changes in microglia following CCI injury. The schematic (**A**) illustrates the workflow of morphological analysis of 3D-reconstructed microglia in the ipsilateral and contralateral cortex. From representative images of 3D-reconstructed microglia from ipsilateral cortex: histograms indicate the ramification index (RI) (**B**), number of branches (**C**), total branch length (**D**), spanned area (**E**), number of junctions (**F**), and number of endpoints (**G**) of 3D-reconstructed microglia. Black dots within bars represents the values from individual 3D-reconstructed microglia (n = 20–25 cells/animal) (n = 5 to 8 animals/group); Two-way ANOVA with Dunnett's multiple comparisons test, error bars representing mean ± SEM; ***p < 0.001, ****p < 0.0001, SHAM or CCI + 3-MP (Low) or CCI + 3-MP (High) versus CCI + Vehicle. Scale bar = 50 μm

Morphological analysis: To evaluate the effects of 3-MP on CCI-induced morphological changes, we analyzed microglial morphology using the motiQ ImageJ plugin (Fig. 9A). Microglia from mice subjected to CCI exhibited significant changes in several morphological features, including the ramification index, number of branches, total branch length, spanned area, number of junctions, and number of endpoints, as compared to sham controls (for all parameters: p < 0.0001), indicative of an ameboid morphology (Fig. 9B–G). Both low and high doses of 3-MP partially but significantly returned these morphological alterations towards sham control levels (for all parameters: p < 0.0001). Specifically, 3-MP treatment restored the ramification index, increased the number of branches and total branch length, expanded the spanned area, and increased the number of junctions and endpoints, indicating a shift-back of ameboid microglial towards a more ramified morphology (Fig. 9B–G).

### 3-MP ameliorates behavioral deficits induced by CCI

We next assessed the effects of 3-MP on the behavior of mice subjected to CCI, specifically examining activity levels, spatial memory, and motor function. Mice underwent a CCI procedure to simulate TBI on day 0, and behavioral assessments were conducted at various time points: 1 week before TBI (day −7), as well as 1 and 2 weeks after TBI (day + 7, + 14). Either 3-MP (26.47 or 13.23 mg/kg–high and low dose, respectively) or vehicle was administered to mice at both 30 min and 24 h post TBI. At 2 weeks after CCI, mice were euthanized for histological and immunostaining analyses (Fig. 6).

**Fig. 10:**
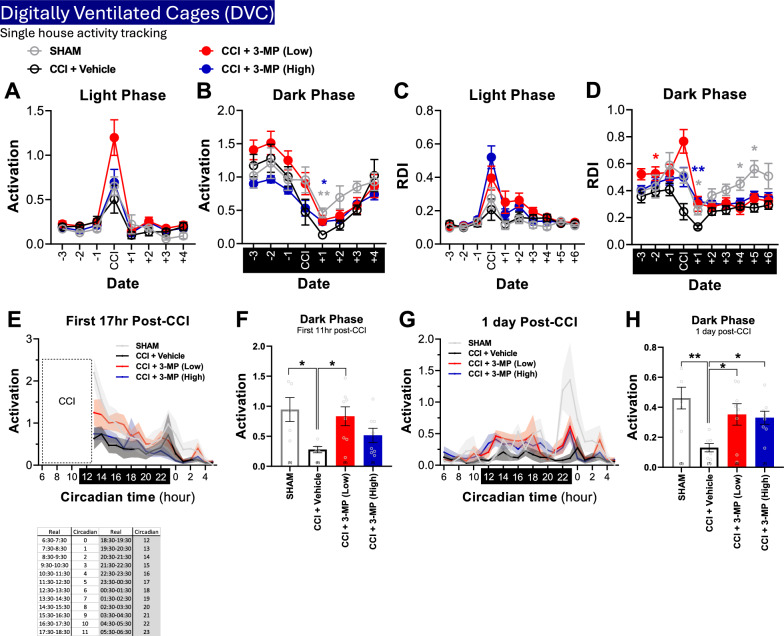
3-MP treatment ameliorates altered cage activity one day following CCI injury. Day to day average activation (**A**, **B**) and Regularity Disruption Index (RDI) (**C**, **D**) during light (**A**, **C**) and dark (**B**, **D**) phases; Mixed effect analysis with Dunnett's multiple comparisons test; *p < 0.05, **p < 0.01, CCI + 3-MP (High) versus CCI + Vehicle (blue), CCI + 3-MP (Low) versus CCI + Vehicle (red), SHAM versus CCI + Vehicle (grey). **E** Home cage activation 17 h after CCI, with each data point representing averaged 2 h blocks of cage activation. Each circadian time point was indicated in the worksheet below. **F** Total activation of dark phase during 11 h after CCI. **G** Home cage activation 1-day post-CCI. **H** Total activation of dark phase 1-day post-CCI. One-way ANOVA with Dunnett’s multiple comparisons test, error bars representing mean ± SEM; *p < 0.05, **p < 0.01, SHAM or CCI + 3-MP (Low) or CCI + 3-MP (High) versus CCI + Vehicle. n = SHAM (5), CCI + Vehicle (7), CCI + 3-MP (Low) (8), CCI + 3-MP (High) (8)

*Home cage activity:* The home cage activity of individually housed mice was monitored prior to and post CCI injury by capacitive sensors placed underneath each cage (Fig. 10). Home cage activity during the light phase showed variability across different dates, especially on the date of CCI. There was an increase in activity across all groups immediately following the CCI or sham procedures during daylight (Fig. 10A). However, on the subsequent day after CCI, home cage activation significantly decreased across all groups. Notably, both sham mice (p < 0.01) and those treated with high dose 3-MP (p < 0.05) exhibited significantly higher home cage activity, as compared to the CCI + vehicle group (Fig. 10B).

Irregularities within the activity pattern of mice, particularly related to rest/sleep-related disturbances, can be quantitatively captured by use of a novel digital biomarker, referred to as a Regularity Disruption Index (RDI). A high RDI is indicative of a more irregular activity rhythm, whereas a low RDI denotes a more consistent/regular pattern. We observed an elevated RDI on the day following the CCI procedure (Fig. 10C), and a substantial decrease in RDI was evident on the day of as well as one day after the CCI procedure during the night circadian (Fig. 10D). A more detailed examination of the timing of activity after the CCI procedure revealed that mice in the sham and 3-MP low dose CCI groups exhibited the most significant increase in home cage activity during the initial half of the dark phase (Fig. 10E). Activity levels of CCI mice in their home cages during the initial 11 h following the CCI/sham procedure showed a significant decrease compared to the sham group (p < 0.05) and the activity of the 3-MP low-dose treatment group was notably higher than that of the control CCI + vehicle group (p < 0.05). Additionally, there was an observed trend in drug efficacy in the 3-MP high-dose + CCI treatment group, although it did not reach statistical significance (Fig. 10F). Twenty-four hours later (night + 1), the control CCI + vehicle mice exhibited lower activity levels, as compared to Shams (p < 0.01) (Fig. 10B, G-H). This effect was significantly mitigated in both the 3-MP + CCI low-dose (p < 0.05) and high-dose (p < 0.05) treated mice when compared to the control CCI + vehicle group (Fig. 10H).

**Fig. 11:**
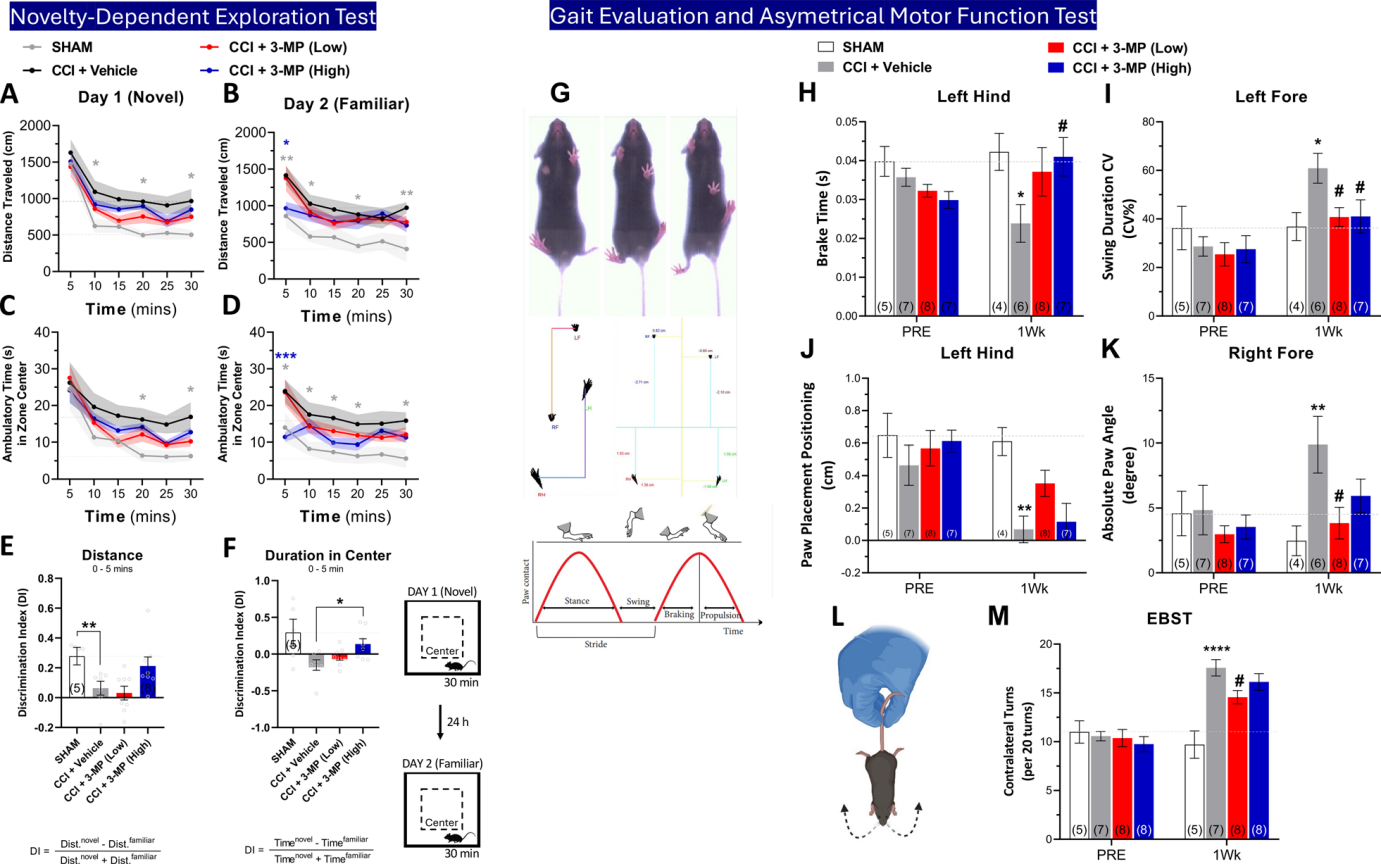
3-MP treatment reduces CCI-induced novelty-dependent exploration behavioral changes, gait impairments, and asymmetrical motor function one week after injury. Distance traveled (**A**, **B**) and ambulatory time spent in the central zone (**C**, **D**) of open field exploration within 30 min were recorded on 2 consecutive days at one week after CCI. Testing on day 1 (**A**, **C**) represents a novel environment for mice w/wo injury, and day 2 as a familiar environment (**B**, **D**); Two-way ANOVA with Dunnett's multiple comparisons test, error bars shown with area filled color representing mean ± SEM; *p < 0.05, **p < 0.01, ***p < 0.001, SHAM (grey) or CCI + 3-PM (High) (blue) versus CCI + Vehicle. The difference between day 1 and day 2 for the distance traveled (**E**) and duration in the center zone (**F**) during the 5 min was assessed by discrimination index; One-way ANOVA with Dunnett's multiple comparisons test, *p < 0.05, **p < 0.01, SHAM (grey) or CCI + 3-PM (High) (blue) versus CCI + Vehicle. (**G**) Representative images were collected by DiGi gait system (Mouse Specifics, Inc.) for gait function assessment. Parameters included brake time (time duration of the initial paw contact to maximum paw contact) (**H**), swing duration CV (% of the coefficient of variance was calculated by 100 X standard deviation/mean, the variability normalized to the mean) (**I**), Paw Placement Positioning (the extent of overlap between ipsilateral fore and hind paws during full stance, indicating the balance metric) (**J**), absolute paw angle (**K**), and were analyzed one week before (PRE) and after CCI (1 Wk). Mouse numbers for each group are indicated at the bottom of bars within the graph. Diagram (**L**) depicts the elevated body swing test (EBST). Asymmetrical function was measured by EBST pre- and post-CCI (**M**). Two-way ANOVA with Dunnett’s multiple comparisons test, error bars representing mean ± SEM; *p < 0.05, **p < 0.01, ****p < 0.0001, SHAM versus CCI + Vehicle; ^#^p < 0.05, CCI + 3-MP (Low) or CCI + 3-MP (High) versus CCI + Vehicle. n = SHAM (5), CCI + Vehicle (7), CCI + 3-MP (Low) (8), CCI + 3-MP (High) (8)

The novelty-dependent exploration test is used to assess memory function in mice by evaluation of their ability to differentiate between a new and familiar environment 24 h after initial exposure. The initial locomotor response observed is, in part, dependent on mice recognizing their presence in a new environment. We observed a substantial decrease in the overall distance traveled (Fig. 11B) and the time spent in the center zone (Fig. 11D) of the open field chamber during the 30-min test in sham mice on the second day (Day 2) of the test, as compared with the data obtained from day 1 (Fig. 11A, C). This reduction was particularly notable during the initial 5 min of the trial. In contrast, control CCI + vehicle mice appeared not to recognize the same open field chamber on the second day (Day 2) of the test, as they exhibited a similar level of traveled distance (Fig. 11B) and time spent within the center zone (Fig. 11D), as compared to their performance on the first day (Day 1) (Fig. 11A, C). Additionally, both sham and high-dose 3-MP CCI mice demonstrated significantly lower distance traveled (Fig. 11B) and ambulatory time in the center zone (Fig. 11D), compared to the control CCI + Vehicle mice during the initial 5 min of the trial on Day 2.

We utilized the discrimination index (DI) to assess behavioral differences between two consecutive days, particularly during the initial 5 min of the trial (Fig. 11E, F). A significant decrease in the DI of distance traveled was observed for control CCI + vehicle mice, as compared to the sham group (p < 0.01) (Fig. 11E). Notably, the high-dose 3-MP CCI group displayed a substantially higher DI of distance traveled compared to the control CCI + Vehicle group, which had a value not significantly different from the sham (p = 0.729) (Fig. 11E). Moreover, the high-dose 3-MP CCI mice exhibited a significantly higher DI of duration in the center, as compared to the control CCI + Vehicle mice (p < 0.05) (Fig. 11F).

*Gait analysis*: Alterations in natural gait patterns were quantified by DigiGait software (Fig. 11G). A significant decline in brake time (Fig. 9H) (p < 0.05 vs. sham) and paw placement positioning (Fig. 11J) (p < 0.01 vs. sham) was evident in CCI + vehicle mice, as compared to the sham group, in the left (contralateral) hind limb at a week after injury. Treatment with high dose 3-MP significantly alleviated this CCI-induced reduction in brake time (p < 0.05) (Fig. 9H), whereas low dose 3-MP demonstrated a trend towards improvement (Fig. 11H, J). Moreover, substantial increases in the coefficient of variation (CV) of swing duration (Fig. 11I) (p < 0.05 vs sham) and paw angle (Fig. 11K) (p < 0.01 vs sham) were noted in the CCI + vehicle group, as compared to sham, in relation to the left (contralateral) and right (ipsilateral) forelimbs, respectively. 3-MP at both low and high-doses (p < 0.05 vs CCI + Vehicle) significantly reduced this CCI-induced elevation of swing duration CV, and low dose 3-MP mitigated the paw angle impairment (p < 0.05 vs CCI + Vehicle) (Fig. 11K).

*Elevated body swing test*: The assessment of asymmetrical motor function was evaluated by EBST (Fig. 11L). Analysis before CCI (PRE) revealed no baseline differences across groups. In contrast, a significant increase in asymmetry behavior was evident in the CCI + vehicle group, as compared to the sham group (p < 0.0001) (Fig. 11M), at 1 week post injury. Treatment with low dose 3-MP notably reduced this CCI-induced asymmetry (p < 0.05 vs CCI + Vehicle), with the high dose exhibiting a trend towards normalization, compared to the control CCI + vehicle group (Fig. 11M).

## Discussion

TBI stands as a significant cause of mortality and prolonged disability at any stage of life, affecting individuals across all age groups [69, 70], with a substantially higher prevalence in males. For survivors, it can result in immediate debilitation and pose challenges that endure for years, particularly among individuals experiencing recurrent head injuries [71]. Consequently, TBI is commonly perceived as a process that unfolds over time. Initially, it comprises a focal brain deformation as the primary injury, followed by ensuing secondary processes that include neuroinflammation, oxidative stress, and an excitotoxicity response [72]. Neuroinflammation is instigated by, and very rapidly appears alongside the initial TBI, and may persist for an extended time after a single injury. It constitutes an essential element of the brain's primary defense system critical in the initiation of reparative processes to restore homeostasis. Even so, prolonged or excessively heightened levels of inflammatory proteins can augment damage, and lead to secondary injury. Unchecked, this can trigger cascades that ultimately result in accelerated neurodegeneration and development of chronic traumatic encephalopathy [73, 74]. A higher prevalence of PD and AD has been associated with TBI [75], and may result from the triggering of attendant gene pathways [12, 75]. Currently, there are no US FDA approved medications designed for treating TBI, despite extensive investigation of multiple different drug classes targeting diverse mechanisms associated with TBI. There is hence an urgent current need for an effective TBI pharmacological treatment. Emerging evidence indicates that neuroinflammation is a significant contributor to neurodegenerative conditions [18–21, 27, 76–79], and, although classical anti-inflammatory agents have been evaluated and have largely failed in TBI clinical trials [80, 81], immunomodulatory drugs that regulate inflammatory signaling via multiple parallel mechanisms offer new promise [27, 37]. In the present study, the novel IMiD 3-MP that potently binds human cereblon but lacks downstream actions on SALL4, effectively decreased pro-inflammatory cytokines following an inflammatory challenge in cell culture and in the brain of mice challenged with LPS as well as TBI—resulting in mitigation of key motor and mnemonic behavioral parameters.

Our prior studies to evaluate the ability of available clinically approved IMiDs to mitigate inflammation, and particularly neuroinflammation, in neurodegenerative disorders have primarily focused on pomalidomide [33, 35, 37]; a third-generation thalidomide analog. Thalidomide, although lowering TNF-α generation in cellular and animal studies, induced dose-limiting adverse events in a human Phase 2a AD study that precluded its administration at desired predetermined doses predicted to target CNS efficacy [82], and hence would be unlikely to be effective in human TBI. In contrast, the second generation more potent and better tolerated IMiD, lenalidomide, although in a current clinical trial for neurodegeneration [83], is a substrate for the efflux transporter p-glycoprotein [84] that expels drugs from brain and, hence, achieves relatively low CNS levels (CSF/plasma ratio: 0.11 [85]); this makes it unlikely to be efficacious in human TBI. Yet more potent, pomalidomide has a brain/plasma concentration ratio of 0.8, and has demonstrated efficacy in preclinical models of PD, TBI and AD [33, 35, 37, 86]. All three clinically approved IMiDs, however, induce teratogenic actions via their binding with human cereblon, the targeting protein within the DDB1–Cul4–Rbx1–CRBN (CRL4 CRBN) E3 ubiquitin ligase complex [37–42]. Their binding to human cereblon induces a conformational change that results in the recruitment of a different series of neosubstrates and their subsequent degradation. Amongst these newly targeted proteins are the zinc finger transcription factors SALL4 and Aiolos [37–42, 60]. As noted, human SALL4 gene mutations associate with developmental disorders [56–58] that share key facets with thalidomide embryopathy [59], whereas cellular Aiolos levels underpin the anticancer activity classical IMiDs [42, 60, 61].

In a quest to develop better tolerated IMiDs, we synthesized IMiD analogs that do and do not bind to human cereblon [27, 37, 53, 62, 87]. This binding interaction is largely mediated via the glutarimide ring of pomalidomide, lenalidomide and thalidomide that interacts with several invariant cysteine and tryptophan residues within a shallow surface thalidomide-binding domain within the C-terminal portion of human cereblon [38, 39, 41, 42]. The glutarimide moiety inserts into a hydrophobic pocket comprising three tryptophan residues (tri-Trp; W380, W386, and W400) and one cysteine residue (C412) to allow the remaining phthalimide ring to extend outward from the surface of cereblon to support interactions with key neosubstrates [38, 39, 41, 42]. Our generation of 3,6′-DTP provided a novel IMiD that potently binds human cereblon but does not result in the downstream degradation of SALL4 [35, 37]. Although pharmacologically active in mouse models of TBI and AD [33–35, 37], 3,6′-DTP proved to be short-lived in mouse and human plasma, as evident in Fig. 4A. This thereby, focused our interest on 3-MP—a close analog and potential metabolite which potently binds human cereblon (Fig. 1A, B) but, does not induce the rapid time-dependent downstream degradation of neosubstrates [SALL4 and Aiolos (Fig. 1C1, C2. D1, D2)] that associates with pomalidomide/thalidomide cereblon binding. Interestingly, as evaluated by molecular modeling, the alignment and key amino acid interactions between 3-MP and the human cereblon/DDB1/SALL4 complex substantially mirrored that of pomalidomide (Fig. 2), albeit the length and strength of the interactions may differ for 3-MP versus pomalidomide with the protein complex. In this regard, computed potential interactions between pomalidomide and target amino acids were poorer in comparison to those for 3-MP (Residue-wise Energy Calculations and Jaguar calculations), with 3-MP forming potential van der Waals and Coulombic interaction with E377, H378, W380, and C412 (predicted as more than − 3 kcal/mol). Additionally, hydrogen bonding to thiocarbonyl groups (C = S) generally differs from that of carbonyl groups (C = O) in several ways, including bond lengths, angles, and overall hydrogen-bonding patterns that support different interactions with target proteins. This potentially underpins some of the differences between barbiturates and thiobarbiturates [88], and is the main difference between 3-MP and pomalidomide. Notably, however, the resulting degradation of SALL4 proved to be markedly different between the two compounds (Fig. 1C1), and the mechanistic basis of this remains a focus of current studies. The biological relevance of this was evaluated in early developmental stage chicken embryos exposed to 3-MP over a range of concentrations, and no adverse development anomalies were observed at any concentration tested. This indicates 3-MP is not teratogenic in chicken embryos under the conditions evaluated. Chicken embryos are valuable models to study development and utilize the same signalling pathways and developmental processes as in humans, making them suitable to screen drugs to gain insight into potential actions in humans [89]. Moreover, chicken embryos have been used to screen thalidomide and analogs of thalidomide to determine their actions and molecular signalling events for many decades [31, 90–94]. Indeed, cereblon’s role in thalidomide teratogenicity was discovered, in part, using chicken embryos [39].

Significantly 3-MP possesses anti-inflammatory activity in cell culture (Fig. 3) and in mouse (Fig. 5) following LPS challenge, and proved to be relatively stable in plasma in ex vivo and in vivo studies (Fig. 4A, B)—achieving a brain/plasma concentration ratio of 0.44 to 0.47 (Table 2). This data aligns well with a recent 3-MP rat study that reported a brain/plasma concentration ratio of 0.51 to 0.61 [64], and the compound proved relatively stable in ex vivo plasma and liver homogenate samples derived from rats and humans [64]. The current study administered 3-MP by the I.P. route across pharmacokinetic, LPS challenge and TBI evaluations. Clearly, however, the drug can be administered via the intravenous and oral routes [64], which are more in line with human use, and the measured oral bioavailability of 3-MP is 38.5% [64] and supports the choice of different administration routes. Furthermore, 3-MP conforms well to the Lipinski rule of 5 and has a CNS MPO (multiparameter optimization) score of 5.4 and calculated Log P value (cLogP: 0.077) that compare favorably to thalidomide and pomalidomide (Table 3), and are in accord with a neurologically active drug [27].

**Table 3:** Computed drug-like properties of 3-MP in comparison to IMiD analogues, thalidomide and pomalidomide

Compound	cLogP value	MPO score
Thalidomide	0.016	5.8
Pomalidomide	− 0.163	4.8
3-Monothiopomalidomide (3-MP)	0.077	5.4

Given the pivotal role of neuroinflammation in driving the progression and secondary injury post-TBI [18–25, 73–75], we characterized the anti-neuroinflammatory effects of 3-MP. Employing an in vivo CCI-induced mouse model of TBI, we evaluated whether 3-MP could effectively modulate neuroinflammatory responses and reduce the subsequent morphological and functional alterations in glial cells—specifically astrocytes and microglia—associated with TBI. Astrocytes are critically involved in maintaining central nervous system homeostasis and their response to injury, astrocyte activation, often assessed by GFAP expression, serves as a hallmark of neuroinflammation [95]. Astrocytes transition from a quiescent to a reactive state in response to TBI, where they can either support neuronal survival or contribute to neurodegeneration, depending on the balance of pro-inflammatory and reparative signals [96, 97]. In the current 3-MP CCI study, we observed a marked increase in GFAP^+^ astrocytes in the ipsilateral cortex post-CCI, indicative of a heightened neuroinflammatory response. Notably, treatment with 3-MP significantly reduced the number and mean intensity of GFAP + cells, suggesting an effective attenuation of astrocyte activation. These findings align with prior research indicating the potential of IMiDs to modulate the inflammatory response within the CNS [86, 87, 98, 99]. The mitigation of astrocyte activation by 3-MP could be attributed to several mechanisms. Firstly, 3-MP may inhibit the production or signaling of pro-inflammatory cytokines, which are known to contribute to astrocyte activation [100]. Pro-inflammatory cytokines such as TNF-α, IL-1β, and IL-6 are critically involved in the pathogenesis of TBI and are potent activators of astrocytes [101]. By reducing levels of these cytokines, 3-MP could limit the extent of astrocyte activation and subsequent neuroinflammatory damage. Additionally, 3-MP may enhance the expression of neurotrophic factors or cytokines involved in CNS repair and regeneration, thereby supporting a more neuroprotective role for astrocytes [102]. These findings are significant as they suggest that 3-MP not only reduces the neuroinflammatory response but may also promote a more conducive microenvironment for neuronal recovery and repair. This is particularly relevant given the lack of effective treatments for TBI and the failure of many classical anti-inflammatory agents in clinical trials for TBI [80, 81, 103]. In addition to GFAP expression, a detailed morphological analysis of astrocytes demonstrated significant changes in the features of astrocytes following the CCI injury, including modifications of surface area, cell volume, branch area, branch length, and total number of branches. These morphological alterations are indicative of reactive astrogliosis, whereby astrocytes become hypertrophic with increased GFAP expression and altered process complexity [104]. Treatment with both low and high doses of 3-MP significantly mitigated these morphological alterations, restoring astrocyte morphology. This suggests that 3-MP not only reduces astrocyte activation but also preserves or restores normal astrocytic architecture, which is critical for maintaining CNS homeostasis and facilitating recovery after injury [105].

Given the intricate interplay between astrocytes and microglia in the context of neuroinflammation, it is equally important to consider the impact of 3-MP on microglial activation and morphology. Microglia, the primary immune cells of the CNS, rapidly respond to brain injury by transitioning from a ramified, surveillant state to an amoeboid, phagocytic phenotype [106]. This activation is associated with the release of pro- and anti-inflammatory cytokines and other mediators that may either exacerbate or manage neuronal damage. Therefore, assessing microglial response provides insight into the neuroinflammatory milieu post-TBI, and potential therapeutic effects of 3-MP. We assessed microglial activation and morphological changes, using IBA1, a calcium-binding protein that is reliably upregulated in activated microglia [107]. We observed elevated expression of IBA1 in microglial cells following CCI injury, which was effectively abated by 3-MP. We also observed significant changes in key morphological features of microglia from CCI mice, including a reduced ramification index, number of branches, total branch length, spanned area, number of junctions, and number of endpoints, compared to sham controls. These changes are indicative of a shift towards a more amoeboid, activated microglial phenotype [108]. Both low and high doses of 3-MP partially reduced these measures. This is in line with the immunomodulatory properties of IMiDs, and with previous results [27, 33–37, 53, 62, 64, 66, 86, 87]. Together with the observed cytokine-modulating effect, results suggest that 3-MP affected the functional phenotype of microglia, promoting the transition from an inflammatory to an anti-inflammatory state. Altogether, our study provides strong evidence that 3-MP can modulate both astrocyte and microglial activation and morphology following TBI.

Whereas an inflammatory response is an essential instigator of neuro-reparative mechanisms following TBI [19–21], in excess it can trigger critical pathological processes that not only contribute to immediate cellular and tissue damage but also impact long-term functional outcomes. In this regard, we quantitatively evaluated a broad array of behavioral outcomes that included mnemonic cage activity (Fig. 10) and novelty-dependent exploration (Fig. 11) as well as motor features [gait impairment and asymmetric motor function (Fig. 11)]. In this regard, human subjects with TBI commonly experience sleep disturbances that impact their quality of life [109]. We hence employed an automated home cage monitoring system to quantify irregular activity patterns (RDI values) potentially linked to sleep and rest disturbances. Changes in RDI have been noted across several disease models beyond TBI, including amyotrophic lateral sclerosis [49] and myotonic dystrophy type 1 [110]. In our study, we observed that the control CCI mice consistently exhibited a lower RDI (particularly in the dark phase [111]) than sham mice, which was substantially mitigated by 3-MP. Memory impairment often results from moderate to severe TBI [112, 113], and was evaluated by a novelty-dependent exploration test in our studies. This demonstrated a lack of recognition of a familiar environment in control CCI vs. sham mice that also was mitigated by 3-MP. Gait impairments are a common feature of TBI in both preclinical models and the human disorder, and associate with injury severity [114–118]. These were evident in our study and significantly ameliorated by 3-MP. The modulation of neuroinflammatory responses, as observed with 3-MP treatment, provided a mechanism through which motor and cognitive improvements were achieved in TBI challenged mice. Chronic neuroinflammation is known to impair neurogenesis, synaptic plasticity and neuronal function, impacting reparative processes and leading to a slower recovery from TBI-induced cognitive deficits and behavioral changes [119–121].

Our study has several caveats. The short duration of our dosing regimen (two doses over 24 h) and evaluation period (14 days post dosing) makes our preliminary safety evaluation short-term and strictly limited. Clearly, a wider range of 3-MP doses, administered over a longer duration, with a focused pathological evaluation of biological tissues is essential in future studies. Our study, as the first in mouse 3-MP investigation, focused on males. This choice was made to evaluate whether 3-MP possessed anti-inflammatory action and potential efficacy in a well characterized TBI preclinical model by using the least number of mice possible. Sex-related disparities have been noted in relation to a variety of brain insults, including TBI [122–124] with outcomes altering with age, menopausal status, TBI severity and selected measures. Although rodent studies generally report poorer outcomes in males versus females, in human investigations the reverse often is described [122], with the presence/absence of hormones such as estrogen being a potential confound. Nevertheless, having gained a clear signal of activity in male mouse studies, future ones necessitate evaluating efficacy in females to characterize potential sex differences in both pharmacokinetic and pharmacodynamic parameters, as well as to identify potential biomarkers of 3-MP responses. Notably, although the tolerability of 3-MP in the chicken embryo assay is promising, future teratogenicity evaluation in other species, such as the rabbit and nonhuman primate [89, 92], is imperative to support clinical translation.

In synopsis, unlike other IMiDs such as thalidomide and lenalidomide, which have shown limited CNS availability and adverse effects, 3-MP exhibits a favorable profile in terms of CNS penetration and short-term safety, making it a promising candidate for further preclinical investigation [123]. In this regard, individuals who survive TBI manifest considerable short- and long-term functional and cognitive challenges for which safe and effective clinically approved pharmacological treatment is lacking. A disproportionate inflammatory response appears to perturb reparative mechanisms and drive pathological processes. The IMiD drug class has demonstrated efficacy across animal models of TBI and neurodegenerative disorders [21, 27, 33, 34, 37, 62] to mitigate inflammation and improve outcome measures, and a new generation of IMiDs that appear not to result in the degradation of key neosubstrates associated with the classical toxicity of this drug class represents a promising avenue for drug development [40]. 3-MP has encouraging pharmacological features and thus warrants further investigation as a candidate drug for TBI and neurodegenerative disorders associated with a prominent neuroinflammatory component.

## Conclusion

The novel IMiD 3-MP potently binds to human cereblon but does not trigger the degradation of key neosubstrates, in particular SALL4, in a manner similar to pomalidomide and thalidomide that associate with adverse actions. 3-MP nevertheless maintains potent immunomodulatory effects to regulate the anti-inflammatory response both systemically and within the brain. This action, in large part, underpinned its ability to mitigate TBI-induced functional impairments. Possessing drug-like properties suitable for clinical translation, 3-MP warrants further preclinical evaluation as a candidate to treat neurological and systemic disorders driven by an over excessive inflammatory response.

## Supplementary Information

Additional file 1:** Supplemental Figure S1. **Docking poses of crystal structure of human cereblon in complex with DDB1 and Lenalidomide. **A** 3-D interactions of lenalidomide with interacting amino acids of human cereblon complex; **B** 2-D interactions of lenalidomide with interacting amino acids of human cereblon complex; **C** 3-D interactions of 3-MP with interacting amino acids of human cereblon complex and **D** 2-D interactions of 3-MP with interacting amino acids of human cereblon complex. **Supplemental Figure S2.** Docking poses of human cereblon in complex with SALL4 and-thalidomide. **A** 3-D interactions of-thalidomide with interacting amino acids of human cereblon complex; **B** 2-D interactions of-thalidomide with interacting amino acids of human cereblon complex3-D interactions of 3-MP with interacting amino acids of human cereblon complex and2-D interactions of 3-MP with interacting amino acids of human cereblon complex. **Supplemental Table S1.** The re-docking of human cereblon PDBs against their native ligands with RMSD evaluation. **Supplemental Table S2.** Docking score of 3-MP with all PDB entries following removal of their native ligands.

## Data Availability

All data and materials are available on reasonable request to the authors.
