# Supplementary material for: Targeting neuroinflammation: 3-monothiopomalidomide a new drug candidate to mitigate traumatic brain injury and neurodegeneration
Source: J Biomed Sci. 2025 Jun 16;32:57. doi: 10.1186/s12929-025-01150-w (PMC12172326; doi:10.1186/s12929-025-01150-w)
Supplement: Supplementary file 1 — Additional file 1: Supplemental Figure S1. Docking poses of crystal structure of human cereblon in complex with DDB1 and Lenalidomide. A 3-D interactions of lenalidomide with interacting amino acids of human cereblon complex; B 2-D interactions of lenalidomide with interacting amino acids of human cereblon complex; C 3-D interactions of 3-MP with interacting amino acids of human cereblon complex and D 2-D interactions of 3-MP with interacting amino acids of human cereblon complex. Supplemental Figure S2. Docking poses of human cereblon in complex with SALL4 and-thalidomide. A 3-D interactions of-thalidomide with interacting amino acids of human cereblon complex; B 2-D interactions of-thalidomide with interacting amino acids of human cereblon complex3-D interactions of 3-MP with interacting amino acids of human cereblon complex and2-D interactions of 3-MP with interacting amino acids of human cereblon complex. Supplemental Table S1. The re-docking of human cereblon PDBs against their native ligands with RMSD evaluation. Supplemental Table S2. Docking score of 3-MP with all PDB entries following removal of their native ligands. [file 12929_2025_1150_MOESM1_ESM.docx]

**Supplemental Information**

**Chemical characterization: 3-Monothiopomalidomide (3-MP)**

**3-(4-amino-1-oxo-3-thioxoisoindolin-2-yl)piperidine-2,6-dione**: ^1^H-NMR (400 MHz, DMSO-*d_6_*): δ 11.08 (s, 1H, NH), 7.62 (s, 2H, NH_2_), 7.46-7.41 (m, 1H, 6-H), 7.13 (d, *J* = 8.4 Hz, 1H, 7-H), 6.98 (d, *J* = 4.8 Hz, 1H, 5-H), 5.63-5.38 (m,1H, 3’-H), 2.90-2.40 (m, 3H, 4’-1H, 5’-2H) and 1.97-1.95 (m, 1H, 4’-H) ppm. Anal. Calcd for C_13_H_11_N_3_O_3_S: C, 53.97; H, 3.83; N, 14.52; S, 11.08. Found: C, 53.93; H, 3.83; N, 14.39; S,11.11.


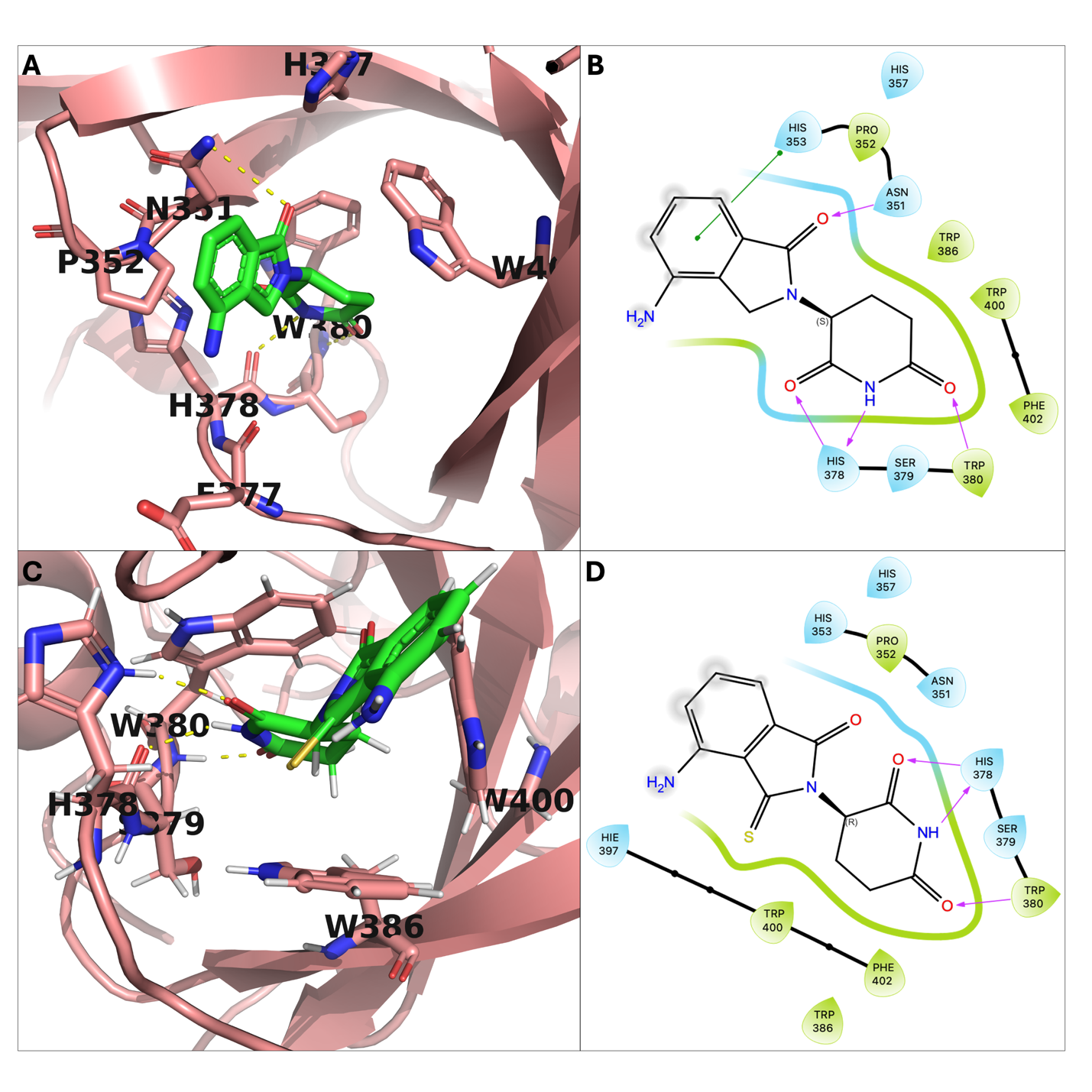


**Supplemental Figure 1. Docking poses of crystal structure of human cereblon in complex with DDB1 and Lenalidomide (PDB ID:4TZ4).** (A) 3-D interactions of lenalidomide with interacting amino acids of human cereblon complex; (B) 2-D interactions of lenalidomide with interacting amino acids of human cereblon complex; (C) 3-D interactions of 3-MP with interacting amino acids of human cereblon complex and (D) 2-D interactions of 3-MP with interacting amino acids of human cereblon complex.

**
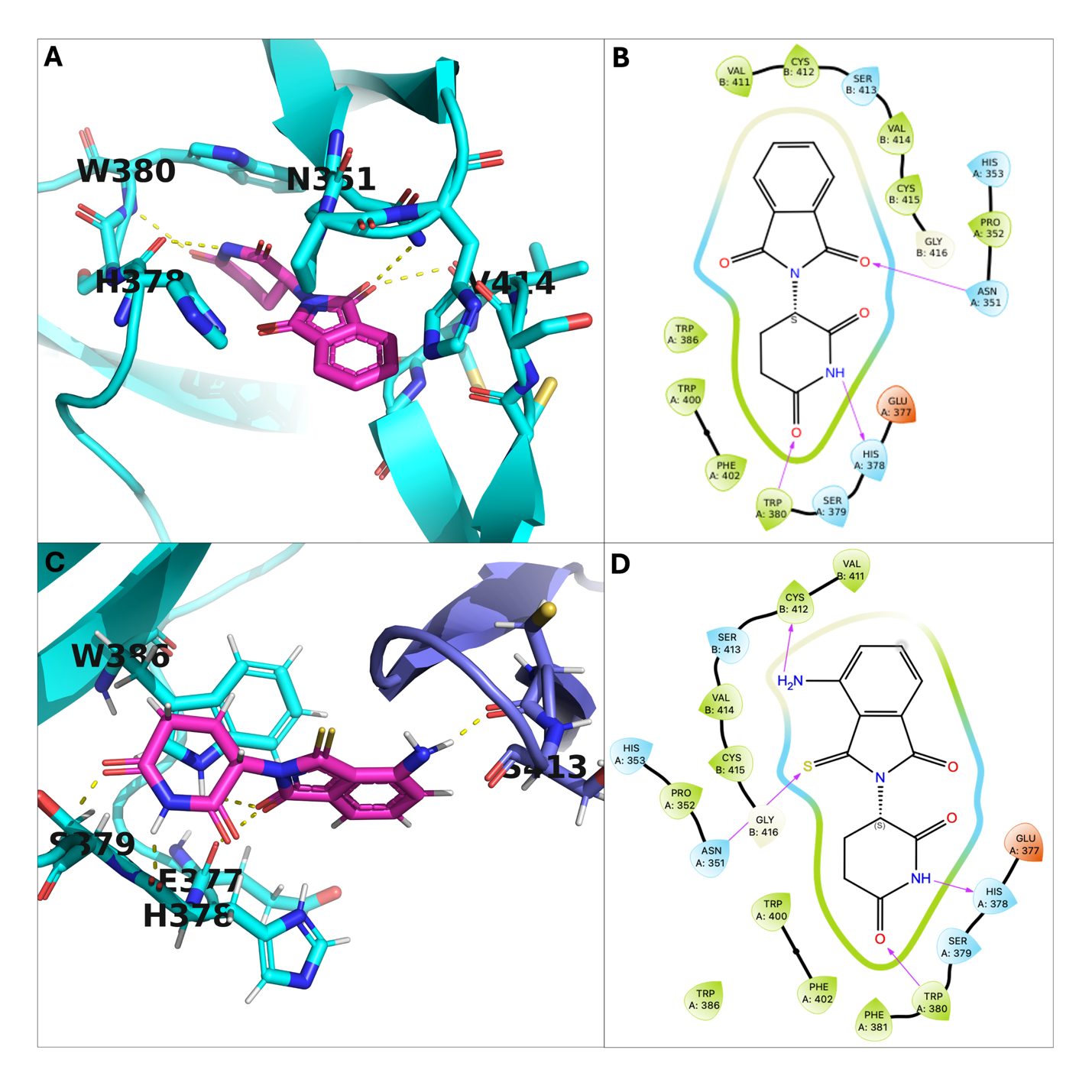
**

**Supplemental Figure 2. Docking poses of human cereblon in complex with SALL4 and (S)-thalidomide (PDB ID:7BQU).** (A) 3-D interactions of (S)-thalidomide with interacting amino acids of human cereblon complex; (B) 2-D interactions of (S)-thalidomide with interacting amino acids of human cereblon complex (C) 3-D interactions of 3-MP with interacting amino acids of human cereblon complex and (D) 2-D interactions of 3-MP with interacting amino acids of human cereblon complex.

**Supplemental Table 1. The re-docking of human cereblon PDBs against their native ligands with RMSD evaluation.**

| **Sr. No.** | **PDB entries with Native ligands** | **Docking score**  **(kcal/mol)** | **RMSD (Å)** |
| --- | --- | --- | --- |
| **1** | **4TZ4- Lenalidomide** | -10.892 | 0.1256 |
| **2** | **6UML-Pomalidomide** | -12.954 | 0.1415 |
| **3** | **7BQU -(S)-thalidomide** | -10.945 | 0.1995 |

**Supplemental Table 2. Docking score of 3-MP with all PDB entries following removal of their native ligands.**

| **Sr. No.** | **PDB entries** | **Docking score**  **(kcal/mol) of 3-MP** |
| --- | --- | --- |
| **1** | **4TZ4** | -11.69 |
| **2** | **6UML** | -12.47 |
| **3** | **7BQU** | -12.17 |
